# Dominant Somatotype Development in Relation to Body Composition and Dietary Macronutrient Intake among High-Performance Athletes in Water, Cycling and Combat Sports

**DOI:** 10.3390/nu16101493

**Published:** 2024-05-15

**Authors:** Marius Baranauskas, Ingrida Kupčiūnaitė, Jurgita Lieponienė, Rimantas Stukas

**Affiliations:** 1 Faculty of Biomedical Sciences, State Higher Education Institution Panevėžys College, 35200 Panevėžys, Lithuania; ingrida.kupciunaite@panko.lt (I.K.); jurgita.lieponiene@panko.lt (J.L.); 2 Department of Public Health, Institute of Health Sciences, Faculty of Medicine, Vilnius University, 01513 Vilnius, Lithuania; rimantas.stukas@mf.vu.lt

**Keywords:** body composition, cycling sports, combat sports, elite athletes, nutrition, somatotype, water sports

## Abstract

Relevant properties of the somatotype as important indicators can be associated with the body composition characteristics as well as both metabolic and bio-mechanical efficiency of athletes in the sport concerned. The primary aim of this single cross-sectional study was to determine the somatotype profiles in association with body composition and nutritional profiles among Lithuanian elite athletes (*n* = 189) involved in water, cycling and combat sports. The body composition along with the somatotype profiles and the nutritional status of athletes were evaluated using a battery of multiple frequency (5, 50, 250, 550, and 1000 kHz) bioelectrical impedance analysis (BIA) and a 3-day food record analysis. In terms of the prediction for athletes to be classified as endomorphs, mesomorphs or ectomorphs, the linear discriminant analysis was conducted to assess the grouping of samples. Both the multiple linear regression and multivariate logistic regression statistical analyses were performed to explore the associations between the independent and dependent variables. The central tendency values for the somatotype components of endomorphy, mesomorphy and ectomorphy in athletes playing water, cycling and combat sports were 4.3–4.9–3.4, 4.3–4.8–3.4 and 4.5–5.5–2.9, respectively. The central mesomorph somatotype with a trend towards endomorphy was dominant and varied according to a high muscle-to-fat ratio in elite athletes. Significant (*p* ≤ 0.001) positive associations between both endomorphy and mesomorphy values and higher body fat percentage as well as lower and upper limb muscle mass were identified. The lower levels of trunk muscle mass were related to athletes’ endomorphy and mesomorphy, too. Furthermore, in the athletes’ sample under analysis, high-level mesomorphs were prone to consume low-carbohydrate (adjusted odd ratio (AOR) 0.5, 95% confidence interval (CI) 0.2; 0.9) and high-protein diets (AOR 2.5, 95% CI 1.1; 5.5). Contrastingly, the elite athletes with a higher expression of endomorphy were on high-carbohydrate (AOR 5.4, 95% CI 1.1; 8.3) and high-fat diets (AOR 4.6, 95% CI 1.5; 7.1) along with insufficient protein diet (AOR 0.3, 95% CI 0.1; 0.9). Finally, whilst nutrition goals as a mediator can play a significant role in undergoing the maintenance of balance between the optimal body composition for athletic performance and the development of an ecto-mesomorphic somatotype, the elite athletes with higher levels of endomorphy value should be aware of lowering the body fat percentage coupled with dietary fat reduction and higher protein intakes. The findings obtained from the study may serve as an antecedent for a more targeted management of the elite athletes’ training process. Somatotyping as an additional assessment method can be successfully deployed in choosing correct coaching techniques, contributing to talent recognition processes or identifying reference morphometric parameters in elite athletes competing in water, cycling and combat sports.

## 1. Introduction

A somatotype is defined as a quantitative expression of the morphological conformation formed of three components which, according to the predominant shape and composition of the human body, are classified into endomorphy, mesomorphy and ectomorphy [1]. Based on the theoretical framework proposed by Heath and Carter [2], the physical properties of the human body are not directly assigned to a particular somatotype. Therefore, each individual has a specific ratio of the three body types related to a mix of endomorphy, mesomorphy and ectomorphy which is also mediated by genetic traits as well as environmental factors [3,4,5,6,7,8,9].

Throughout an athlete’s development, anthropometry plays a significant role in the regular selection of athletes and their performance measures. Evidently, the somatotype assessment is particularly helpful in sports where the body type may affect the improvement of movement performance [10,11]. Generally, the comparison of somatotypes of sportsmen competing in the Olympics with referent cohorts revealed that high-performance athletes are more mesomorphic and less endomorphic [12]. However, some anthropometric studies have also found diversities in somatotypes among athletes depending on their participation in different sports. The mesomorphic element was pre-dominant in sports depending on speed and strength, for instance, combat sports, weightlifting, rowing and swimming [13,14]. In the case of high jumpers, their somatotype was a balanced ectomorph [13], while the ecto-mesomorphic somatotype proved to be the most appropriate body type for athletes competing in cyclic sports [15].

Furthermore, relevant properties of each somatotype as important indicators can be associated with health-related anthropometric characteristics [16] and both, metabolic and biomechanical efficiency of athletes in the sport concerned [4]. For example, the endomorphic element was related to higher levels of fat mass, the mesomorphic component was associated with increased muscle mass, and the ectomorphic unit was related to a body height-weight ratio [17,18]. In addition, various athletes engaging in different sports change their body constitution characteristics like body weight and segmental proportions, especially those of the lower and upper limbs [11,19]. Taking into account that the dynamics of the development of a specific body shape depending on sport-specific training plays a key role in elite athletes from different sports, relevant somatotype studies and anthropometric measurements of the estimated body segment parameters are needed and necessary.

Additionally, there is some evidence on the relationship between the body type and the nutritional status. For example, previous studies carried out by Tanner et al. [20] and Gordon et al. [21] revealed an association between endomorphy and higher serum cholesterol concentrations. The nutritional benefits of the average daily consumption of macronutrients were emphasized. Therefore, the nutritional profile is likely to be associated with the dominant somatotype; however, each somatotype relies on different dietary requirements, types of exercise, and refueling after hard workouts. Given that endomorphs are more sensitive to the hormone insulin and the intake of carbohydrates [22], they are recommended to use a low-glycemic-index diet in order to control blood glucose levels. They should also be aware of an adequate intake of proteins. Furthermore, mesomorphs are typically athletic with an increase in muscle fiber and can induce muscular hypertrophy along with increased levels of body fat more easily compared to ectomorphs. Therefore, mesomorphs are suggested for consuming high-protein foods [23]. Since ectomorphs have a tendency to more intensive lipolysis, they reduce fat mass easily; therefore, ectomorphic subjects are recommended a high-carbohydrate diet and combine evenly divided portions of protein and fat [24]. Nevertheless, there are only limited data [25] on exploring the association between the somatotypes and the intake of nutrients among high-performance athletes.

Considering that the scientific literature reveals a diverse approach to the potential impact of somatotypes on success in sport, it is also known that predisposition is only one of the components which, together with other factors such as the training process, nutritional status, psychological preparation, plays a key role in a successful career of athletes meaning ‘an athlete is born and created’ [20]. Although it is obvious that an anthropometric profile may refer to the athletes’ suitability to engage in high-performance sport [26,27,28,29,30,31,32,33], there is an existing research gap in explaining the relationship between the body composition profile, nutritional status and the quantified expression of the morphological conformation in elite athletes. Therefore, the transition between somatotypes and their practical implications in sports should be explored at a more advanced level.

This study was conducted in order to determine the components of somatotypes in association with body composition and nutritional profiles among Lithuanian elite athletes involved in water, combat and cycling sports. To achieve the research objective, the subsequent alternative hypotheses (H) were constructed.

**H_1_.**  *The core components of the body composition have an association with the three main components of the somatotype in elite athletes.*

**H_2_.**  *The dietary macronutrient intakes have a relation to somatotype profiles in a cohort of elite athletes.*

## 2. Materials and Methods

### 2.1. Study Design and Subjects

The study was conducted as a single cross-sectional study. In the one-year period of 2019–2020, all elite athletes were pooled and selected on a voluntary basis in agreement with the list approved by the Lithuanian National Olympic Committee (LNOC). The main inclusion criteria for athletes were set as follows: (1) elite athletes exercising during a preparatory phase; (2) attendees of the European and World Sports Championships; (3) applicants for Lithuania’s Olympic Team (LTeam). The target clusters of elite athletes engaging in water, cycling and combat sports amounted to 81, 51 and 62 potential study participants. The representative sample size with a confidence level of 95% and a marginal error of 2% was calculated via OpenEpi software version 3.01 [34] and equaled to 79, 50 and 61 for each cluster of athletes competing in water, cycling and combat sports, respectively. Therefore, during the preparatory phase, the observational study recruited 189 elite athletes with a mean age of 18 ± 3.8 years who represented the candidates to LTeam and participated in water sports (*n* = 79), cycling sports (*n* = 51) and combat sports (*n* = 59).

### 2.2. Measures

All the measurements of target athletes were carried out during a standard medical examination at the Lithuanian Sports Medicine Centre (LSMC). The body composition along with somatotype profiles and nutritional status of athletes were evaluated using a battery of bioelectrical impedance analysis (BIA) [35] and a 3-day food record analysis (3-DFRA) [36,37].

#### 2.2.1. Body Composition Assessment

BIA as a widely-used [38] and rapid (≤5 min) third level of validity [35,39] method was applied to estimate the body composition of athletes. More specifically, the X-scan instrument (International Organization for Standardization adopted by the European Union (EN-ISO): 13488, Seoul, Republic of Korea) via sending multi-frequency currents of 5, 50, 250, 550, and 1000 kHz through the body for greater penetration of different tissues [40,41] was used to estimate the size of the main body composition components such as body weight, total body water, lean body mass, muscle mass, trunk muscle mass, lower and upper limb muscle mass, total body protein, mineral, and body fat mass in kilograms (kg) and percentages (%). In addition, height was assessed to the nearest 0.1 cm using a stadiometer which was integrated as part of the BIA device. The accuracy of the body composition assessment was optimized when athletes followed specific pre-testing guidelines: (1) without eating for 8 h; (2) without drinking for 2 h; (3) without exercising for 24 h; (4) without alcohol consumption for 48 h; (5) emptying the bladder/bowels within 30 min before testing.

#### 2.2.2. Somatotype Assessment

As shown in Table 1, the outcomes obtained from the BIA led to calculating the values of the main somatotype components (endomorphy, mesomorphy, ectomorphy) by using the valid equations which were established by Bertuccioli et al. [42]. As a consequence, the elite athletes were categorized depending on the somatotype profiles by using the Heath–Carter method [1].

In terms of ‘low’, ‘moderate’, ‘high’ and ‘very high’ levels of endomorphy, mesomorphy and ectomorphy, the calculated values were able to range between 0.5 and 2.5, 2.5 and 5, 5 and 7 as well as ≥7, respectively [1]. Moreover, it should be highlighted that the estimated values gave the magnitude of each of the three somatotype components. For example, if a rating of 4.1–4.9–2.5 was recorded, endomorphy was rated as 4.1, mesomorphy was considered to be 4.9, and ectomorphy was graded as 2.5. Overall, six somatotype profiles of athletes could be recognized based on body type ratings in that fashion [46]: (1) ‘endomorphic mesomorph’ (mesomorphy was dominant and endomorphy exceeded ectomorphy by more than 0.5 units; (2) ‘balanced mesomorph’ (mesomorphy predominated, and both endomorphy and ectomorphy were analogous or the difference between these ones was not greater than 0.5 units); (3) ‘ectomorphic mesomorph’ (mesomorphy was dominant and ectomorphy exceeded endomorphy by more than 0.5 units; (4) ‘mesomorph-ectomorph’ (endomorphy and ectomorphy were analogous or the difference between those was not greater than 0.5 units), while endomorphy was smaller; (5) ‘mesomorphic ectomorph’ (ectomorphy was dominant and mesomorphy was greater by 0.5 units than this component value compared to endomorphy; (6) ‘balanced ectomorphy’ (ectomorphy predominated, and endomorphy and ectomorphy were analogous or the difference between these ones was not greater than 0.5 units).

#### 2.2.3. Nutritional Status Assessment

The self-reported 3-day physical activity recall was obtained at the same time when the athletes recorded all of the foods and beverages they had eaten and drunk [36,37] on the basis of the amounts of foods contained in the ‘Atlas of Foodstuffs and Dishes’ [47]. The analysis of energy expenditure and macronutrient intakes was performed by a sports dietician from LSMC. During the following data-processing phase, a list of athletes’ average daily food consumption was established as well as the daily intakes of carbohydrates, protein and fat were calculated using NutriSurvey software (http://www.nutrisurvey.de/, accessed on 25 April 2024) (SEAMEO-TROPMED RCCN-University of Indonesia) [48] in which the function ‘Food/Include more foods from other databases’ was extended as the nutrition values of food items were manually integrated from the Lithuanian food database [49]. The daily intakes of dietary macronutrients were compared to the reference daily intakes (RDIs) for carbohydrates (5–8 g/kg/day and 45–55% of energy intake (EI)), protein (1.2–2.2 g/kg/day and 15–20% of EI) and fat (0.8–1.5 g/kg/day and 25–35% of EI) as reported by the International Society of Sports Nutrition (ISSN) [50,51]. The EI (kcal/day and kcal/kg/day) of athletes was further calculated taking into account the consumption of dietary macronutrients. The EI was compared to the daily energy expenditure (DEE) which was estimated by totaling several components, namely, the basal metabolic rate (BMR) and training energy expenditure (TEE) [52]. In addition, the Harris–Benedict equation [53] was applied to estimate BMR. The physical activity codes along with metabolic equivalents (METs) (kcal/kg/h) for physical activities reported by Ainsworth et al. [54] were used to calculate both, TEE and DEE.

### 2.3. Statistical Data Analysis

The statistical data analysis was conducted using the Statistical Package for the Social Sciences (IBM SPSS Statistics) version 25.0 for Windows (IBM Corp, Armonk, NY, USA). The graphic representation of data obtained from the study was performed via SPSS software. The Shapiro–Wilk test was used to test the normality of the study data. The relative frequency tables were used to represent all categorical data. The measures of central tendency, namely, means (*M*s) ± standard deviations (*SD*s) were calculated for each variable. Both, the independent *t*-test and the analysis of variance (ANOVA) were applied to assess the differences between the means of the body composition characteristics, energy and dietary macronutrient intakes, and the magnitude of somatotype components. Additionally, for post hoc multiple comparison procedures, the significance of the differences between the group means was assessed using Tukey’s honestly significant difference (HSD) test [55]. Furthermore, the one-sample *t*-test was applied to estimate whether there was a significant difference between the grouped measures of central tendency (the size of the body composition components vs. reference values; energy and macronutrient intakes vs. RDIs). The results obtained following the one-sample *t*-test were coupled with Cohen’s D (*d*) estimates served as the effect sizes. In line with Cohen [56], the results were interpreted as follows: ‘a small effect size’ (0.2 ≤ *d* < 0.5), ‘a moderate effect size’ (0.5 ≤ *d* < 0.8), and ‘a large effect size’ (*d* ≥ 0.8). Also, the magnitude of the mean differences between the body composition characteristics following ANOVA was expressed with the standardized effect size (*η*^2^*_p_*). The cut-offs for qualitative descriptors of *η*^2^*_p_* were interpreted as follows: ‘small effect size’ (0.01 ≤ *η*^2^*_p_* < 0.06), ‘a medium effect size’ (0.06 ≤ *η*^2^*_p_* < 0.14), and ‘a large effect size’ (*η*^2^*_p_* ≥ 0.14).

In terms of the prediction for athletes to be classified as endomorphs, mesomorphs or ectomorphs, the linear discriminant analysis (LDA) was performed to assess the grouping of samples [57]. The LDA was performed via the stepwise method and the model was constructed step by step. Specific variables allowing the discrimination between the groups were incorporated into the analysis. At the end of the analysis, the percentage of correctly classified cases was calculated.

Finally, the multiple linear regression models were obtained to assess the association between the somatotype profiles as the dependent variables and the core components of body composition. The multiple linear regression models were adjusted for the sex and age of athletes. Moreover, the multivariate logistic regression analyses were performed to calculate the adjusted odds ratios (AORs) and 95% confidence intervals (CIs) as well as to reveal whether a relationship persists between the somatotype expression in athletes (dependent variables) and the intakes of carbohydrates, protein and fat (independent variables). The dependent variables, namely, endomorphy, mesomorphy and ectomorphy were converted to the dichotomous forms as follows: (a) ‘0’—endomorphy value < 5 (reference category) and ‘1’—endomorphy value: from 5 to 7; (b) ‘0’—mesomorphy value < 5 (reference category) and ‘1’—mesomorphy value: from 5 to 7; (c) ‘0’—ectomorphy value < 3 (reference category) and ‘1’—ectomorphy value: ≥3. All the multivariate logistic regression models were adjusted for athletes’ age and type of exercise.

In all the statistical tests used in data analysis, the critical value of the significance level was set as α = 0.05.

## 3. Results

### 3.1. Characteristics of Athletes

In the sample under analysis, the athletes were engaged in a mix of aerobic and anaerobic sports (*n* = 95) and aerobic sports (*n* = 95). Based on more detailed data, the athletes participated in sports activities as follows: (a) water sports: boat racing (*n* = 24), canoe paddling (*n* = 12) and swimming (*n* = 43); (b) cycling sports: track cycling (*n* = 11) and road cycling (*n* = 40); (c) combat sports: boxing (*n* = 14), taekwondo (*n* = 4), Graeco-Roman wrestling (*n* = 29) and judo (*n* = 12). The study sample consisted of 74.1% male athletes (*n* = 140) and 25.9% female athletes (*n* = 49). The average career expectancy of athletes was 8.0 ± 3.8 years. The elite athletes exercised moderately for 168 ± 53.2 min per day and 6 days a week. More detailed information on the characteristics of elite athletes is provided in Table 2.

### 3.2. Anthropometric Profiles and Nutritional Status

Although physiological sex differences may lead to disparities in the size of body composition core elements [58,59], Table 3 provides the study results revealing the differences between anthropometric characteristics in the samples of male and female athletes who competed in different sports activities.

**Figure 1:**
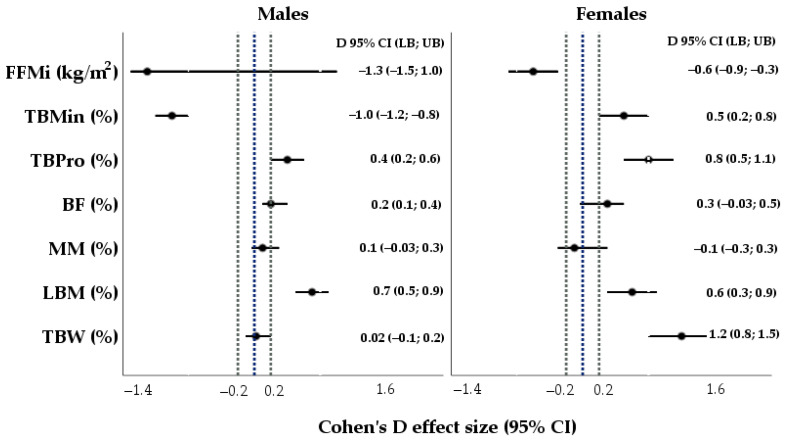
The effect size (Cohen’s D) was used to indicate the standardized difference between the means of anthropometric characteristics and the reference values for body composition in the cohorts of both male and female athletes. TBW—total body water (ref. value: for M—60%, for F—52.5%); LBM—lean body mass (ref. value: for M—80%, for F—75%); MM—muscle mass (ref. value: for M—77%, for F—72%); TBPro—total body protein (ref. value: for M—17%, for F—15%); TBMin—total body mineral (ref. value: for M—5.9%, for F—5.7%); BF—body fat (ref. value: for males—15.5%, for females—21.5%); FFMi—height-adjusted fat-free mass index (ref. value: for males—20.8%, for females—17.6%). Dotted blue line means reference value for body composition. Dotted gray lines represent thresholds beyond which the effect sizes are significant. 95% CI—95% confidence interval; ref.—reference; LB—lower bound; UB—upper bound; M—males; F—females.

Figure 1 shows a more in-depth analysis of the study data that referred to a relatively higher lean body mass (in %), muscle mass (in %) and a lower body fat percentage among LTeam male athletes involved in water, cycling and combat sports and at the same time corresponded to better body composition prognoses for elite athletes. Meanwhile, the body composition was less optimal for elite sports depending on excessive body fat percentage in the group of female athletes (*d* 0.3, 95% CI −0.03; 0.5). 

Taking into account energy intake, as displayed in Table 4, the mean energy (47 ± 16 kcal/kg/day) of athletes was slightly below the target RDI (52 ± 8 kcal/kg/day) (*d* −0.3, 95% CI −0.2; −0.5). Irrespective of sports type, both a significant deficit of carbohydrate intake (5.5 ± 2.3 g/kg/day; *d* −0.4, 95% CI −0.6; −0.3) and overconsumption of fat (39.2 ± 7.1%; *d* 1.3, 95% CI 1.1; 1.5) were observed in athletes’ nutrition. No statistically significant difference was found in terms of the profile of athletes’ protein intake (1.7 ± 0.6 g/kg/day; *d* −0.1, 95% CI −0.2; 0.1) compared to RDI (1.2–2.2 g/kg/day).

### 3.3. Magnitude of Somatotype Components

**Figure 2:**
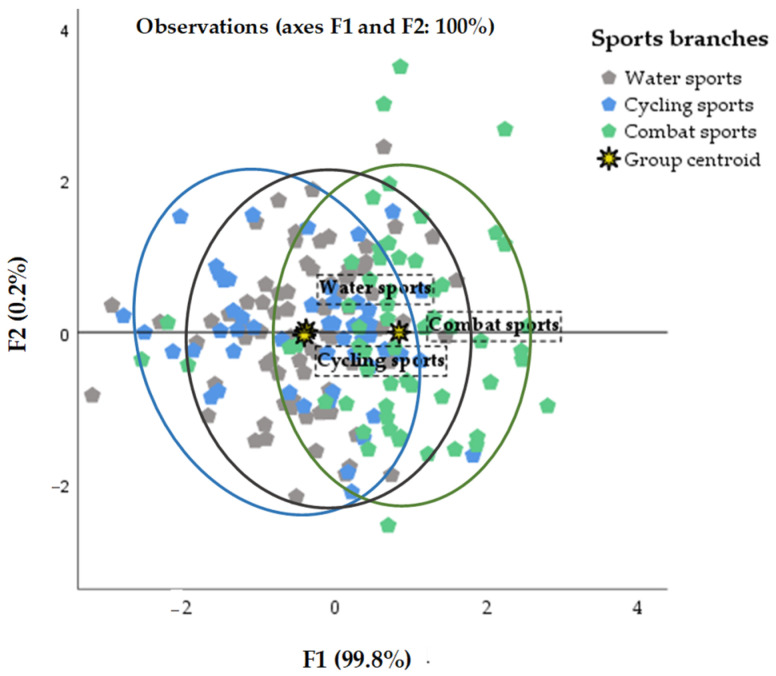
The scatter plot displaying confidence ellipses of the first two discriminant functions obtained from the stepwise linear discriminant analysis of somatotypes (mesomorph and endomorph) pre-domination according to athletes’ sports branches (water sports, cycling sports and combat sports). F1—function 1; F2—function 2.

Figure 2 shows the results of the stepwise discriminant function analysis referring to a prosperous prediction of athletes’ capability to be classified into the elite cohort which can be attained using higher values of both mesomorphy and endomorphy. As for the athletes’ groups competing in water, cycling and combat sports, the chances of 83.5%, 84.3% and 71.2% for being in the group of endomorphic mesomorphs were calculated, respectively.

As shown in Table 5, the mesomorph body type as the most dominant component was identified in elite athletes representing all sports disciplines. The central tendency values for somatotype components of endomorphy, mesomorphy and ectomorphy in athletes playing water, cycling and combat sports were 4.3–4.9–3.4, 4.3–4.8–3.4 and 4.5–5.5–2.9, respectively.

In addition, following the post hoc multiple comparison analysis, significantly (*p* < 0.05) highest values of both, mesomorphy and endomorphy, were found to be specific to the athletes participating in sports activities such as Graeco-Roman wrestling (5.7 ± 0.6 and 4.6 ± 0.5) and canoe paddling (5.4 ± 0.7 and 4.7 ± 0.4) and at the same time were higher than those referring to track cyclists’ mesomorphy (4.6 ± 0.5) and endomorphy in both, swimmers (4.1 ± 0.4) and road cyclists (4.2 ± 0.4). Meanwhile, the mean ectomorphy values for swimmers and boxers ranged from 3.5 to 3.6 and were significantly (*p* < 0.05) higher when compared to the ectomorphic index fluctuation from 2.5 to 2.7 among elite athletes competing in sports such as canoe paddling, taekwondo and Graeco-Roman wrestling (Table 5).

### 3.4. Somatotype in Association with Body Composition and Nutritional Status

**Figure 3:**
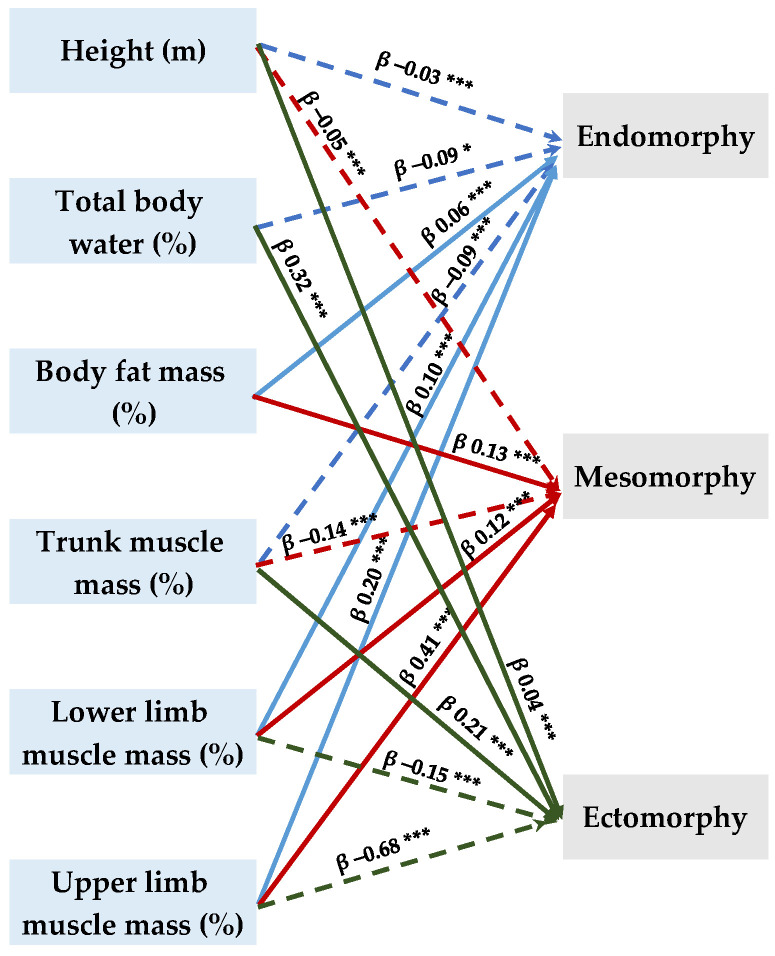
A graphic representation of multiple linear regression models (independent variables: athletes’ height (m), total body water (%), body fat mass (%), trunk muscle mass (%), lower limb muscle mass (%), and upper limb muscle mass (%); dependent variables are the types of somatotypes: endomorphs, mesomorphs, and ectomorphs. The multiple regression models were adjusted for the sex and age of athletes. A dotted line means negative association. *—*p* ≤ 0.05, ***—*p* ≤ 0.01. See Table A1 for further details.

Following the adjustment for sex and age, multiple linear regression models were performed to verify the associations between the magnitude of somatotype components (endomorphy, mesomorphy, ectomorphy) as dependent variables and the main components (height, total body water, body fat mass, trunk muscle mass, lower and upper limb muscle mass) of athletes’ body composition as independent variables. Figure 3 and Appendix A Table A1 in display significant (*p* ≤ 0.001) positive relationships between both, endomorphy and mesomorphy values, and higher body fat percentage as well as lower limb muscle mass and upper limb muscle mass. Meanwhile, lower levels of trunk muscle mass were related to the athletes’ physical types referring to endomorphy and mesomorphy, respectively. Furthermore, the athletes’ sample under analysis revealed a positive association of moderate values of ectomorphy and height, trunk muscle mass and a negative correlation with upper and lower limb muscle mass.

**Figure 4:**
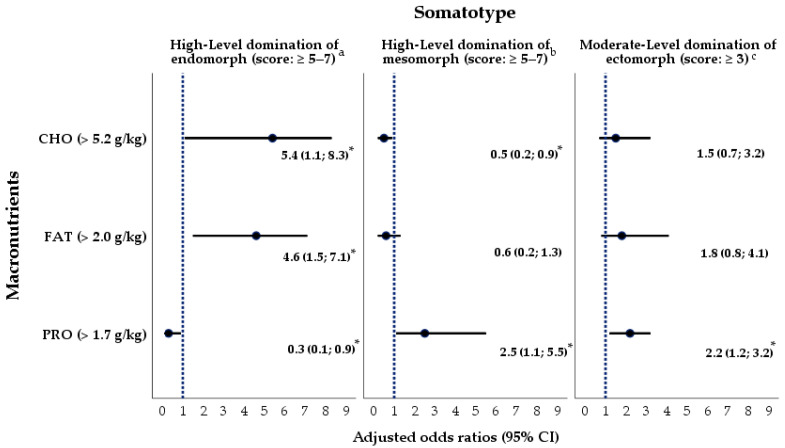
A graphic representation of multivariate logistic regression models (^a–c^) (dependent variables: the somatotypes of athletes: endomorphs, mesomorphs, and ectomorphs; independent variables: the intake (g/kg of body weight per day) of macronutrients, namely, carbohydrates, proteins, and fat). A dotted line means that the adjusted odds ratio (AOR) is equal to 1. If AOR > 1 and AOR ≠ 1, it means a positive association between the independent and dependent variables. If AOR < 1 and AOR ≠ 1, it means there is a positive association between the independent and dependent variables. The logistic regression models were adjusted for the athletes’ age and type of exercise. See Table A2 for further details. *—*p* ≤ 0.05, 95% CI—95% confidence interval, CHO—carbohydrates, PRO—protein, FAT—fats.

Further multivariate logistic regression analyses related to the predominance of a somatotype and a nutritional status revealed the following significant relationships among elite athletes involved in water, cycling and combat sports (Figure 4). Following the adjustment of logistic regression models for athletes’ age and type of exercise, the AORs for higher intakes of carbohydrates and fat along with the insufficient protein consumption in the subgroup of high-level endomorphs were 5.4 (95% CI 1.1; 8.3), 4.6 (95% CI 1.5; 7.1) and 0.3 (95% CI 0.1; 0.9), respectively. Contrastingly, in the subgroup of athletes with pre-dominant mesomorphy values, the diet was low in carbohydrates (AOR 0.5, 95% CI 0.2; 0.9) and high in proteins (AOR 2.5, 95% CI 1.1; 5.5). Finally, whilst ectomorphs need a diet higher in proteins, our study also found a significant positive association between a moderate level of ectomorphy and a high-protein diet consumed by athletes (AOR 2.2, 95% CI 1.2; 3.2).

## 4. Discussion

### 4.1. Dominant Somatotype Proportion in Athletes

The analysis of somatotypes examined in the cohort of elite athletes showed the dominance of the mesomorphy index which reflected the possible musculoskeletal robustness. This study also identified the disparities in mesomorphy values among athletes competing in different sports. The lowest mesomorphy index of 4.6 was identified in Lithuanian track cyclists. On the contrary, the groups of athletes involved in water and combat sports, namely, canoe paddling and Graeco-Roman wrestling, revealed the highest average values of mesomorphy equalling 5.4 and 5.7, respectively. In our research, the level of mesomorphy expression (5.4) in combat sports athletes was relatively higher compared to the average mesomorphy value (5.0, 95% CI 4.6; 5.4) identified in combat sports athletes (wrestlers, boxers, taekwondists, and judokas) from other countries such as Korea [60], Ukraine [13], Turkey [9,61,62,63], Mexico [64], Germany [65], Malaysia [66], Spain [67], Croatia [68], Columbia [69], Poland [70,71], Montenegro [72], Algerian [73], Brazil [74], India [75] and Uzbekistan [76]. This study, along with the previous study carried out in Lithuania by Gutnik et al. [19], highlighted high values of mesomorphy (5.4 and 6.2) in canoe paddlers exceeding the average mesomorphy index (4.8, 95% CI 4.4; 5.1) found among canoe paddlers representing other countries, namely, Poland [77], India [78], Spain [79] and Britain [80]. Furthermore, in terms of water sports, our study identified similar values of mesomorphy (4.4) in boat racers compared to the average mesomorphy index 4.4 (95% CI 3.9; 4.8) obtained from Turkish [81], Spanish [82], Greek [83], Australian [27], Pakistani [84], Croatian [85] rowers and revealed a significantly lower expression of Lithuanian swimmers’ mesomorphy (4.1) if contrasted with the average mesomorphy index 4.6 (95% CI 3.7; 5.4) which was disclosed in American [86], Ukrainian [13], Malaysian [87], Spanish [88] and Serbian swimmers [17]. Equivalently, the mean mesomorphy value (4.3) of the Lithuanian cyclists was found to be lower than the average mesomorphy index (5.1, 95% CI 4.0; 6.1) of cyclists from Australia [89], Uzbekistan [90], Czech [91], and Mexico [92].

### 4.2. Somatotype and Body Composition

Based on our study results, all Lithuanian elite athletes were endo-mesomorphs. Obviously, in athletic activities, ecto-mesomorphic somatotype is predominant to other body type characteristics in high-performance sports [2]. In line with these findings referring to the relationship between the expression of somatotypes and body composition, the association was revealed between both mesomorphy and endomorphy and body fat percentage in the study of elite athletes. In addition, the components of mesomorphy and endomorphy were not only related to higher levels of limb muscle mass, but also associated with lower levels of trunk muscle mass in elite athletes. Meanwhile, the moderate ectomorphy index was positively related to athletes’ height, trunk muscle mass, and negatively associated with limb muscle masses. Hence, according to our study data, it becomes possible to make a prognosis that can rely on how to adjust training programs for athletes, taking into account not only the characteristics of body composition, but also the expression of somatotypes. More specifically, a significant increase in mesomorphy for canoe paddlers and combat sports athletes may be argued by the demand for eccentric contractions of many synergetic skeletal muscles (in terms of limb muscles) [93,94]. In this case, the training process for boat paddlers and combat sports athletes was covered by strong contractions of limb muscles and potentially resulted in an increase in muscle size [95]; however, workout plans should be complemented by static loads for strengthening the parts of athletes’ trunk (e.g., girth, shoulder). On the contrary, for elite athletes, especially swimmers and cyclists, the lower mesomorphy value was associated with both a relatively underdeveloped muscle mass of limbs and lower fat stores; therefore, the present anthropometric profiles of athletes may result in insufficient static loads [2] serving as a trigger for muscle hypertrophy during the training process.

### 4.3. Somatotype and Nutritional Status

After our study revealed the relationship between the endo- and mesomorphic components and the slightly excessive body fat, there is a possible association between the dietary intake and the corresponding somatotypes in athletes, too. Given that somatotyping places a reliance on the dietary macronutrient intake and the bodily metabolic pathways taking place in the body [24], our study found an association between the dietary intakes and the body type components in elite athletes.

In the studied sample of elite athletes, a more detailed study data analysis affirmed that high-level endomorphs had higher intakes of both carbohydrates and fat. Surprisingly, only one previous scientific study obtained from Raschka and Aichele [96] has also revealed a correlation between endomorphy and lower carbohydrate and fat intakes in a group of students who played sports. However, earlier findings did not uniformly match our study results. In contrast, it must be emphasized that higher expression of endomorphy in humans may lead to an excessive accumulation of body fat depending on the bioconversion of acetyl coenzyme A into malonyl coenzyme A which, following the elongation phase, serves as a substrate for fatty acids synthesis and results in a higher ability to store adipocytes via lipogenesis [24]. Nevertheless, only a few previous research studies reported an association between serum cholesterol and body type in males, but not in females [20,21]. Moreover, endomorphs are recommended to consume carbohydrates more moderately depending on their elevated sensitivity to hormone insulin [22]. Thus, in our case, in order to prevent weight gain, elite athletes having a more expressed endomorphy value and, in general, eating a low-carbohydrate diet can be guided to consume low-fat foods and adopt a high-protein diet.

In this context, it is appropriate to distinguish athletes with a high mesomorph index. Based on our study data, the mesomorphy component had a positive relationship with protein intake and an inverse association with carbohydrate intake among elite athletes. This finding can be explained by the fact that only high-protein food that contains or is in the absence of carbohydrates is adequate for muscle growth following exercise performance (after strength and power training) [50,97,98]. Furthermore, although mesomorphs may induce muscular hypertrophy along with the increased levels of body fat more easily compared to ectomorphs [24], athletes with a high level of mesomorphy should be aware of an adequate intake of high-carbohydrate and low-fat foods for the purpose of triggering musculoskeletal adaptations to endurance training [50]. Furthermore, our study found that a diet higher only in protein was consumed by the athletes with moderate ectomorphy values. In this context, it should be noted that ectomorphs can burn excess calories while moving continuously and stimulate lipolysis and/or inhibit glucose uptake [24]. In the group of ectomorphs, additional studies [99] have also found an increased dependence on glycolytic metabolism following progressive, maximal exercising on a cycle ergometer. On the basis of the above-mentioned research data, we can recommend the consumption of a high-carbohydrate diet and a combination of evenly divided portions of protein and fat for ectomorphic persons [24].

### 4.4. Strengths, Limitations and Future Directions

According to our knowledge, in the Baltic region of Europe, this study was the first performed to have explored the main body type components in association with the body composition and the dietary macronutrient intakes in elite athletes participating in cycling and combat sports. Hence, the results obtained from our research can help optimize diet and exercise plans catered to various somatotypes. The second strength of our study was related to the assessment of the body type and the composition of high-performance athletes in various sports codes that have not formerly been reported on an analogical scale. Thirdly, the results derived from this study displayed that somatotyping may serve as a rough tool for a potential prediction of the optimal body composition for athletic performance. Fourth, although the body type evaluation using outdated/different constitutional schemes is incompetent in offering a possibility to compare the somatotyping outcomes as reported by other senior scholars [2,100,101], the authors of this study found a modern method [42] for calculating the somatotype values of endomorphy, mesomorphy and ectomorphy following the bioimpedancemetry analysis.

This study had some limitations. There was no opportunity to conduct longitudinal research over a period of time depending on its specificity and a relatively small, yet representative, study sample size of the target population. Taking into account that high-performance athletes from other regions of the world may apply different types of exercise and training regimes or rely on the traditional ways of eating, these differences can lead to disparities in body physique patterns. Therefore, the generalized results obtained by our study have a single probability to be extrapolated to cohorts of Eastern European athletes. In addition, since both the body composition and the somatotype profiles of athletes were estimated indirectly from equations, this way of assessment is a widely applied method in scientific research. Also, our study was limited as we were in no capacity to in the sense to establish a cause-and-effect association between independent and dependent variables or analyze the changes in the main components of somatotypes following the design of the experimental study.

Further research may consider functional or motor tests of high-performance athletes in order to disclose their overall anthropological status. This study could be extended with a larger overall sample size and a longitudinal approach should be applied to track the somatotype expression interchanges [82] over time. The selection based on voluntary participation might introduce bias, as it may not represent the entire spectrum of elite athletes; therefore, in terms of sex, age, and individual morphometric markers, the results obtained from our study reflect the need for similar research to be carried out into other type of sports with a wider differentiation of sportsmen, too. Moreover, although BIA stands as a useful appliance for monitoring changes in hydration status and the distribution of fluid throughout the body, the accuracy varies across BIA devices [35]. Therefore, further directions could be related to the use of dual-energy X-ray absorptiometry (DXA) [102] to assess the body composition of professional athletes. Finally, whilst dietary records are often considered as a reference method, weighted food records may provide more precise estimates of consumed portions. Consequently, further research on the dietary intakes of athletes could rely on weighted food records [103].

## 5. Conclusions

The findings of this study focused on possible development trends toward diversities of body type profiles in relation to different types of athletic performance. The central mesomorph somatotype with a trend towards endomorphy was dominant and varied by a high muscle-to-fat ratio in elite athletes competing in water, combat and cycling sports. These phenomena may result from a combination of professional training and progress on sport-defined shapes. However, it should be highlighted that the somatotype component of an ectomorphic mesomorph is better suited for good athletic performance compared to other body types. Hence, when the workout plans for canoe paddlers and combat sports athletes could be complemented by static loads for strengthening the parts of athletes’ trunks along with reducing body fat patterns, high-performance swimmers and cyclists should improve their musculoskeletal robustness of lower and upper limbs.

In addition, although the nutritional benefits of daily consumption can be related to all somatotypes, the findings of our study referred to the association between higher intakes of protein and moderate expression of ectomorphy in elite athletes. High-level mesomorphs were prone to consume low-carbohydrate and high-protein diets. Furthermore, high-carbohydrate and high-fat diets along with insufficient protein diets were eaten by high-level endomorphs. Finally, whilst nutrition goals as a mediator can play a significant role in undergoing the maintenance of the balance between the optimal body composition for athletic performance and the development of ecto-mesomorphic somatotype, the elite athletes with higher levels of endomorphy value should be aware of lowering the body fat percentage coupled with dietary fat reduction and higher protein intake.

The findings obtained from this study may serve as an antecedent for more targeted management of the elite athletes’ training process. The somatotyping as an additional assessment method can be applied in combination with other correct coaching techniques, contribute to talent recognition processes or identify reference morphometric parameters in elite athletes competing in water, cycling and combat sports. Future longitudinal studies dealing with monitoring somatotype changes throughout an athlete’s career may shed more light on long-term effects.

## Figures and Tables

**Table 1:** The prediction equations for the assessment of somatotype profiles using the bioelectrical impedance analysis (BIA) estimates.

Equation	Reference
Endomorphy value_Males_ = 10.44 − 0.0297 × H (m) − 0.0683 × TBW (%) + 0.150 × BMi (kg/m^2^)	[42,43]
Endomorphy value_Females_ = 4.313 − 0.0572 × TBW (%) + 0.145 × BMi (kg/m^2^)	[42,43]
Mesomorphy value_Males_ = 11.81 − 0.0524 × H (m) − 0.00725 × Rz (Ω) + 0.230 × BMi (kg/m^2^)	[42,43,44]
Mesomorphy value_Females_ = 8.91 − 0.0589 × H (m) − 0.00395 × Rz (Ω)+ 0.317 × BMi (kg/m^2^)	[42,43,44]
Ectomorphy value_Males_ = −60.25 + 0.188 × H (m) + 0.0146 × Rz (Ω) − 0.350 × TBW (kg) + 0.345 × TBW (%) + 0.4174 × BMi (kg/m^2^) + 0.105 × Ei	[42,43,44]
Ectomorphy value_Females_ = −2.119 + 0.119 × TBW (%) + 0.0778 × MM (%) + 0.244 × BMi (kg/m^2^) − 0.709 × FFMi (kg/m^2^)	[42,43,45]
BMi = BW/H (m)^2^	[43]
FFMi = LBM (kg)/H (m)^2^	[45]

Rz—the resistance representative of the intracellular water volume was calculated for 1000 kHz and was equaled to 314.97 Ω [44]; Ei—the edema index was defined as the ratio of extracellular fluid to total body fluid; FFMi—free fat mass index; BMi—body mass index; TBW—total body water; MM—muscle mass; H—height; LBM—lean body mass; BW—body weight.

**Table 2:** Characteristics of elite athletes.

Variables		*n*	%
Sports branches	Water sports		
	Boat racing	24	12.7
	Canoe paddling (500–2000 m)	12	6.3
	Swimming (50–400 m)	43	22.8
	Cycling sports		
	Track cycling	11	5.8
	Road cycling	40	21.2
	Combat sports		
	Boxing	14	7.4
	Taekwondo	4	2.1
	Graeco-Roman wrestling	29	15.3
	Judo	12	6.3
Types of exercise	The mix of aerobic and anaerobic exercise	95	50.3
	Aerobic exercise	94	49.7
Sex	Male athletes	140	74.1
	Female athletes	49	25.9
The duration of exercise	90–180 min per day	144	76.2
	181–300 min per day	45	23.8
Years of participating in sport	<8 years	110	58.3
	9–20 years	79	41.7

**Table 3:** The body composition characteristics depending on elite athletes’ sex and sports branches.

Variables	Water Sports		Cycling Sports		Combat Sports		*η*^2^*_p_* ^a^	*η*^2^*_p_* ^b^
	Males (*n* = 57)	Females (*n* = 22)	Males (*n* = 31)	Females (*n* = 20)	Males (*n* = 52)	Females (*n* = 7)		
Height (m)	1.8 ± 0.1	1.7 ± 0.1	1.8 ± 0.1	1.7 ± 0.04	1.7 ± 0.1	1.6 ± 0.1	0.34	0.08
Body weight (kg)	81.6 ± 11.5	60.2 ± 9.6	71.1 ± 7.9	60.4 ± 6.8	69.9 ± 17.1	57.2 ± 4.3	0.15	0.02
Total body water (kg)	47.8 ± 5.7	34.2 ± 4.1	42.7 ± 3.8	33.6 ± 3.2	42.2 ± 8.4	31.7 ± 2.0	0.15	0.06
Total body water (%)	58.9 ± 2.9	56.5 ± 3.2	60.3 ± 2.8	55.4 ± 2.6	61.0 ± 3.9	55.4 ± 2.8	0.07	0.04
Lean body mass (kg)	66.6 ± 7.4	46.9 ± 5.7	59.2 ± 5.3	46.3 ± 4.4	58.5 ± 11.7	43.9 ± 2.7	0.16	0.04
Lean body mass (%)	81.9 ± 3.9	78.1 ± 4.7	83.7 ± 3.8	76.9 ± 3.7	84.7 ± 5.5	77.0 ± 3.8	0.07	0.02
Muscle mass (kg)	61.9 ± 6.8	43.4 ± 5.2	55.1 ± 4.9	42.8 ± 4.0	54.4 ± 10.8	40.7 ± 2.6	0.16	0.04
Muscle mass (%)	76.2 ± 4.0	72.6 ± 4.3	77.8 ± 3.8	71.1 ± 3.6	78.9 ± 5.4	71.2 ± 3.8	0.06	0.03
Trunk muscle mass (kg)	31.7 ± 3.7	22.1 ± 2.5	27.5 ± 2.4	21.8 ± 1.7	27.1 ± 4.9	20.6 ± 1.2	0.18	0.06
Trunk muscle mass (%)	38.2 ± 2.5	37.2 ± 4.4	38.9 ± 2.0	36.2 ± 2.3	39.4 ± 3.1	36.0 ± 0.2	0.04	0.03
Lower limb muscle mass (kg)	22.5 ± 2.8	16.2 ± 2.1	20.3± 1.8	15.8 ± 1.9	20.0 ± 4.3	14.7 ± 1.0	0.12	0.05
Lower limb muscle mass (%)	27.7 ± 1.6	27.1 ± 2.7	28.6 ± 1.4	26.3 ± 1.9	28.9 ± 1.8	25.8 ± 1.4	0.09	0.06
Upper limb muscle mass (kg)	8.3 ± 1.1	5.7 ± 0.7	7.4 ± 0.7	5.6 ± 0.5	7.4 ± 1.6	5.4 ± 0.3	0.13	0.04
Upper limb muscle mass (%)	10.2 ± 0.7	9.6 ± 1.0	10.4 ± 0.5	9.2 ± 0.6	10.6 ± 0.7	9.4 ± 0.5	0.07	0.04
Total body protein (kg)	13.9 ± 1.5	9.6 ± 1.1	12.4 ± 1.1	9.5 ± 0.9	12.3 ± 2.3	9.0 ± 0.7	0.16	0.05
Total body protein (%)	17.2 ± 1.1	16.1 ± 1.2	17.6 ± 1.0	15.8 ± 0.9	17.9 ± 1.5	15.8 ± 0.1	0.07	0.03
Total body mineral (kg)	4.7 ± 0.7	3.5 ± 1.5	4.2 ± 0.4	3.5 ± 0.4	4.1 ± 0.9	3.3 ± 0.2	0.15	0.02
Total body mineral (%)	5.8 ± 0.1	5.8 ± 0.1	5.8 ± 0.1	5.8 ± 0.1	5.8 ± 0.1	5.8 ± 0.1	0.02	0.001
Body fat mass (kg)	15.1 ± 4.9	13.4 ± 4.4	11.8 ± 3.5	14.1 ± 3.4	11.4 ± 6.5	13.3 ± 3.0	0.09	0.01
Body fat mass (%)	18.0 ± 4.1	21.7 ± 4.3	16.3 ± 3.8	23.1 ± 3.7	15.2 ± 5.4	23.2 ± 3.7	0.07	0.03
FFMi (kg/m^2^)	18.4 ± 1.5	17.2 ± 0.9	18.2 ± 0.7	16.8 ± 0.7	19.5 ± 1.9	17.2 ± 0.9	0.13	0.05

Data are presented as mean ± standard deviation (*M* ± *SD*); The magnitude of mean differences are expressed with standardized effect sizes. The partial eta squared (*η*^2^*_p_*) is the measure of effect size; ^a^—the *η*^2^*_p_* effect size statistic of the differences between anthropometric characteristics of male athletes competing or training within different types of sports, ^b^—the *η*^2^*_p_* effect size statistic of the differences between anthropometric characteristics of female athletes competing or training within different types of sports; FFMi—fat-free mass index.

**Table 4:** Energy and dietary macronutrient intakes in elite athletes depending on sports branches.

Variables	Water Sports (*n* = 79)	Cycling Sports (*n* = 51)	Combat Sports (*n* = 59)	Total (*n* = 189)	RDI	*d* 95% CI (LB; UB) ^a^
Energy intake (kcal/day)	3492 ± 996	3126 ± 1120	3054 ± 830	3257 ± 999	3687 ± 857	−0.4 (−0.6; −0.3)
Energy intake (kcal/kg/day)	47 ± 14	48 ± 18	47 ± 15	47 ± 16	52 ± 8	−0.3 (−0.5; −0.2)
Carbohydrates (g/kg/day)	5.1 ± 1.9	6.1 ± 2.8	5.5 ± 2.0	5.5 ± 2.3	5–8	−0.4 (−0.6; −0.3)
Carbohydrates (% of EI)	43.2 ± 9.1	50.8 ± 7.6	47.0 ± 7.7	46.5 ± 8.8	45–55	−0.4 (−0.5; −0.3)
Protein (g/kg/day)	1.7 ± 0.6	1.7 ± 0.6	1.6 ± 0.6	1.7 ± 0.6	1.2–2.2	−0.1 (−0.2; 0.1)
Protein (% of EI)	14.9 ± 3.4	14.4 ± 3.1	13.8 ± 2.0	14.4 ± 3.0	15–20	−1.0 (−1.2; −0.9)
Fat (g/kg/day)	2.2 ± 0.8	1.8 ± 0.7	2.0 ± 0.8	2.0 ± 0.8	0.8–1.5	1.0 (0.8; 1.2)
Fat (% of EI)	41.9 ± 8.6	34.8 ± 7.4	39.2 ± 7.1	39.2 ± 7.1	25–35	1.3 (1.1; 1.5)

Data are presented as mean ± standard deviation (*M* ± *SD*); The effect size (Cohen’s D) was used to indicate the standardized difference between the means of dietary macronutrient intakes and the recommended daily intakes (RDIs). ^a^—the *d* effect size statistic of the energy and macronutrient intakes differences compared to RDIs. 95% CI—95% confidence interval; LB—lower bound; UB—upper bound; EI—energy intake.

**Table 5:** The categorization of athletes with different levels of the magnitude of each of the three somatotype components depending on different types of sports.

Variables	Somatotype Component			Somatotype Categories
	Endomorphy	Mesomorphy	Ectomorphy	
Water sports (*n* = 79)	4.3 ± 0.5	4.9 ± 0.7 ***	3.4 ± 0.8 ***	Endomorphic mesomorph
Boat racing	4.4 ± 0.4	4.9 ± 0.6	3.3 ± 0.7	Endomorphic mesomorph
Canoe paddling (500–2000 m)	4.7 ± 0.4 ***	5.4 ± 0.7 ***	2.5 ± 0.6 ***	Endomorphic mesomorph
Swimming (50–400 m)	4.1 ± 0.4 ***	4.7 ± 0.6	3.6 ± 0.7 ***	Endomorphic mesomorph
Cycling sports (*n* = 51)	4.3 ± 0.4	4.8 ± 0.6 ***	3.4 ± 0.6 ***	Endomorphic mesomorph
Track cycling	4.4 ± 0.4	4.6 ± 0.5 ***	3.1 ± 0.5	Endomorphic mesomorph
Road cycling	4.2 ± 0.4 ***	4.9 ± 0.6	3.5 ± 0.5	Endomorphic mesomorph
Combat sports (*n* = 59)	4.5 ± 0.6	5.5 ± 0.7 ***	2.9 ± 1.0 ***	Endomorphic mesomorph
Boxing	4.2 ± 0.6	5.3 ± 0.5	3.5 ± 0.9 ***	Endomorphic mesomorph
Taekwondo	4.7 ± 0.6	5.7 ± 0.7	2.7 ± 0.9 ***	Endomorphic mesomorph
Graeco-Roman wrestling	4.6 ± 0.5 ***	5.7 ± 0.6 ***	2.7 ± 1.1 ***	Endomorphic mesomorph
Judo	4.4 ± 0.7	5.3 ± 1.1	3.0 ± 0.9	Endomorphic mesomorph

Data are presented as mean ± standard deviation (*M* ± *SD*); ***—the Tukey’s HSD corrected *p*-value < 0.001; m—metres.

## Data Availability

Data are available as a part of Supplementary Materials.

## Supplementary Materials

The following supporting information can be downloaded at: https://www.mdpi.com/article/10.3390/nu16101493/s1, Dataset S1: Database used for statistical data analysis.

## Nomenclature

**Term**	**Definition**
AOR	Adjusted odds ratio
ANOVA	Analysis of variance
BIA	Bioelectrical impedance analysis
BF	Body fat
BMi	Body mass index
BMR	Basal metabolic rate
BW	Body weight
CI	Confidence interval
CHO	Carbohydrates
*d*	Cohen’s D effect size
DEE	Daily energy expenditure
3-DFRA	3-day food record analysis
DXA	Dual-energy X-ray absorptiometry
Ei	Edema index
EN-ISO	International Organization for Standardization adopted by the European Union
EI	Energy intake
ISSN	International Society of Sports Nutrition
F	Female
FAT	Fats
FFMi	Free fat mass index
H	Height
*H*	Alternative hypothesis
Kcal	Kilocalorie
Kg	Kilograms
kHz	Kilohertz
LB	Lower bound
LBM	Lean body mass
LDA	Linear discriminant analysis
LNOC	Lithuanian National Olympic Committee
LSMC	Lithuanian Sports Medicine Centre
LTeam	Lithuania’s olympic team
*n*	Number of cases in a subsample
*N*	Total number of cases
*p*	*p*-value
PRO	Protein
m	Metres
M	Male
*M*	Sample mean
MET	Metabolic equivalent
MM	Muscle mass
*R* ^2^	Multiple correlation squared/measure of strength of association
RDI	Reference daily intake
Rz	Resistance
*SD*	Standard deviation
SPSS	Statistical package for the social sciences
TBMin	Total body mineral
TBPro	Total body protein
TBW	Total body water
TEE	Training energy expenditure
Tukey’s HSD	Ttukey’s honestly significant difference
UB	Upper bound
USA	The United States of America

## Appendix A

**Table A1:** Association between the body composition and the type of somatotype (endomorphy, mesomorphy, and ectomorphy) as a dependent variable in a sample of elite athletes (multiple regression analyses).

Model	Independent Variable	β	95% CI (LB; UB)	*p*
Endomorphy component ^a^	Height (m)	−0.03	(−0.03; 0.02)	<0.001
	Total body water (%)	−0.09	(−0.16; −0.02)	0.017
	Body fat mass (%)	0.06	(0.02; 0.09)	0.004
	Trunk muscle mass (%)	−0.09	(−0.11; −0.07)	<0.001
	Lower limb muscle mass (%)	0.10	(0.06; 0.14)	<0.001
	Upper limb muscle mass (%)	0.20	(0.09; 0.32)	0.001
Mesomorphy component ^b^	Height (m)	−0.05	(−0.06; −0.04)	<0.001
	Total body water (%)	0.003	(−0.11; 0.11)	0.961
	Body fat mass (%)	0.13	(0.07; 0.19)	<0.001
	Trunk muscle mass (%)	−0.14	(−0.18; −0.10)	<0.001
	Lower limb muscle mass (%)	0.12	(0.05; 0.18)	<0.001
	Upper limb muscle mass (%)	0.41	(0.23; 0.59)	<0.001
Ectomorphy component ^c^	Height (m)	0.04	(0.04; 0.05)	<0.001
	Total body water (%)	0.32	(0.17; 0.47)	<0.001
	Body fat mass (%)	0.06	(−0.03; 0.14)	0.189
	Trunk muscle mass (%)	0.21	(0.16; 0.26)	<0.001
	Lower limb muscle mass (%)	−0.15	(−0.23; −0.06)	0.001
	Upper limb muscle mass (%)	−0.68	(−0.93; −0.43)	<0.001

The multiple regression models were adjusted for the sex and age of athletes; *p*—*p*-value, 95% CI—95% confidence interval; LB—lower bound; UB—upper bound; ^a^—Model 1: adjusted *R*^2^ = 0.84, F_8,180_ = 127.97, *p* < 0.01; ^b^—Model 2: adjusted *R*^2^ = 0.82, F_8,180_ = 106.92, *p* < 0.01; ^c^—Model 3: adjusted *R*^2^ = 0.74, F_8,180_ = 67.13, *p* < 0.01.

**Table A2:** Multivariate logistic regression analyses with levels of different somatotypes in athletes.

Model	Independent Variable	β (SE)	Wald	*p*	AOR 95% CI (LB; UB)	*R* ^2^ *_Nagelkerke_*
High-Level endomorphy (score: ≥5–7) ^a^	Carbohydrates (≥5.2 g/kg/day)	1.7 (0.9)	5.6	0.048	5.4 (1.1; 8.3)	0.31
	Protein (≥1.7 g/kg/day)	−1.4 (0.8)	5.1	0.050	0.3 (0.1; 0.9)	
	Fat (≥2.1 g/kg/day)	1.5 (0.8)	5.4	0.043	4.6 (1.5; 7.1)	
	The constant	−1.7 (0.9)	5.5	0.042	–	
High-Level mesomorphy (score: ≥5–7) ^b^	Carbohydrates (≥5.2 g/kg/day)	−0.8 (0.4)	5.3	0.038	0.5 (0.2; 0.9)	0.22
	Protein (≥1.7 g/kg/day)	0.9 (0.4)	5.0	0.027	2.5 (1.1; 5.5)	
	Fat (≥2.1 g/kg/day)	−0.5 (0.4)	1.3	0.253	0.6 (0.2; 1.3)	
	The constant	2.4 (0.7)	10.7	0.001	–	
Moderate-Level ectomorphy (score: ≥3) ^c^	Carbohydrates (≥5.2 g/kg/day)	0.4 (0.4)	1.2	0.256	1.5 (0.7; 3.2)	0.26
	Protein (≥1.7 g/kg/day)	0.7 (0.3)	5.2	0.042	2.2 (1.2; 3.2)	
	Fat (≥2.1 g/kg/day)	0.6 (0.4)	2.2	0.141	1.8 (0.8; 4.1)	
	The constant	−3.6 (0.8)	20.5	<0.001	–	

SE—standard error; Wald—the Wald test; *p*—*p*-value; AOR—adjusted odds ratio (AOR = e^β^); 95% CI—95% confidence interval; LB—lower bound; UB—upper bound); *R*^2^*_Nagelkerke_*—the Nagelkerke *R*^2^ statistic; ^a^—Model 1: reference category: a low level of endomorphy (value < 5); ^b^—Model 2: reference category: a low level of mesomorphy (value < 5); ^c^—Model 3: reference category: a low level of ectomorphy (value < 3). The logistic regression models were adjusted for athletes’ age and type of exercise.
