# Supplementary material for: Dominant Somatotype Development in Relation to Body Composition and Dietary Macronutrient Intake among High-Performance Athletes in Water, Cycling and Combat Sports
Source: Nutrients. 2024 May 15;16(10):1493. doi: 10.3390/nu16101493 (PMC11124112; doi:10.3390/nu16101493)
Supplement: Supplementary file 1 [file nutrients-16-01493-s001.zip › nutrients-3007835-supplementary.pdf]

Kodas	Sporto_sak	Sport_brar	Sakos	Lytis	Energ_sana	amzius	Bendra_tre	ugis_cm	kuno_mase
1	2.00	13	1.00	1	2724.34	28.00	120.00	163.30	66.50
5	3.00	6	2.00	1	4349.42	33.00	120.00	188.00	87.30
6	3.00	6	2.00	1	4473.48	25.00	120.00	181.00	90.00
7	3.00	5	2.00	1	4262.02	22.00	180.00	184.00	80.40
8	3.00	5	2.00	1	4286.61	23.00	180.00	186.50	80.80
9	3.00	5	2.00	1	4366.98	20.00	180.00	190.00	80.70
10	3.00	5	2.00	1	4034.27	23.00	180.00	180.00	74.80
11	3.00	5	2.00	1	4074.03	20.00	180.00	182.00	73.90
12	3.00	5	2.00	1	4240.39	20.00	180.00	190.00	76.50
13	3.00	5	2.00	1	4057.00	20.00	180.00	181.00	73.70
13	3.00	5	2.00	1	4002.75	20.00	300.00	181.00	71.90
14	2.00	11	1.00	1	4740.12	31.00	180.00	186.00	100.00
15	3.00	5	2.00	2	2419.13	17.00	150.00	166.00	57.20
16	3.00	5	2.00	2	2475.85	17.00	180.00	168.00	60.30
17	3.00	5	2.00	2	2505.43	17.00	120.00	175.00	60.80
18	3.00	5	2.00	2	2334.30	16.00	150.00	165.00	51.70
19	3.00	5	2.00	2	2459.46	16.00	150.00	167.50	58.90
20	3.00	5	2.00	1	3967.15	17.00	120.00	183.00	68.50
21	3.00	5	2.00	1	3685.20	17.00	90.00	176.00	61.70
22	3.00	5	2.00	1	3735.64	18.00	112.00	174.00	64.60
24	3.00	5	2.00	1	4110.19	18.00	120.00	187.50	72.10
25	3.00	5	2.00	1	4274.69	18.00	120.00	183.00	79.20
26	3.00	5	2.00	1	3818.98	18.00	120.00	175.00	67.00
28	3.00	5	2.00	1	4001.89	19.00	180.00	176.00	73.20
29	3.00	5	2.00	1	3819.62	17.00	180.00	180.00	64.70
30	3.00	5	2.00	1	2977.83	17.00	120.00	158.00	44.80
31	3.00	5	2.00	1	3778.68	16.00	120.00	173.00	65.40
32	3.00	5	2.00	1	3662.89	17.00	120.00	180.00	59.50
33	3.00	6	2.00	2	2529.09	17.00	90.00	171.00	63.00
33	3.00	6	2.00	2	2494.99	18.00	180.00	171.00	61.40
34	2.00	11	1.00	1	3662.08	15.00	120.00	172.00	61.40
37	2.00	11	1.00	1	3757.42	16.00	150.00	176.00	63.60
39	2.00	11	1.00	1	3154.23	15.00	90.00	162.00	48.20
39	2.00	11	1.00	1	3520.42	15.00	120.00	172.00	56.70
41	2.00	11	1.00	1	4513.37	15.00	120.00	185.00	84.90
42	3.00	5	2.00	1	4031.13	19.00	180.00	174.00	74.90
44	2.00	10	2.00	1	4393.42	22.00	240.00	188.00	83.30
47	3.00	4	2.00	1	4796.81	19.00	240.00	191.00	94.10
57	3.00	6	2.00	2	2540.96	16.00	180.00	168.00	63.80
62	3.00	6	2.00	2	2617.97	16.00	180.00	166.50	68.80
63	3.00	6	2.00	2	2620.55	15.00	180.00	169.00	68.00
64	2.00	10	2.00	2	2553.88	26.00	180.00	169.00	69.30
66	3.00	7	1.00	1	3541.82	15.00	240.00	166.00	59.60
67	3.00	7	1.00	1	3994.63	16.00	180.00	174.00	72.20
68	3.00	7	1.00	1	3708.30	16.00	240.00	174.00	62.70
70	3.00	7	1.00	1	3770.10	16.00	180.00	177.70	63.40

71	3.00	7	1.00	1	3570.75	15.00	120.00	170.00	59.10
72	3.00	7	1.00	1	2982.10	14.00	120.00	156.50	44.00
73	3.00	7	1.00	1	4335.74	14.00	90.00	179.00	80.70
74	3.00	5	2.00	2	2429.74	17.00	180.00	170.00	57.10
75	2.00	10	2.00	2	2337.36	16.00	180.00	166.00	51.70
76	2.00	10	2.00	2	2430.32	15.00	120.00	165.50	57.00
77	3.00	5	2.00	2	2486.22	18.00	120.00	174.00	60.30
81	2.00	11	1.00	1	4045.78	18.00	120.00	180.00	72.70
84	2.00	11	1.00	1	4044.77	17.00	120.00	178.00	72.90
89	3.00	5	2.00	1	4205.15	16.00	210.00	183.00	75.90
90	3.00	5	2.00	1	3657.06	18.00	90.00	168.50	64.00
91	3.00	4	2.00	1	4785.18	18.00	150.00	190.50	93.40
92	3.00	4	2.00	1	4693.39	17.00	180.00	193.40	88.80
93	3.00	4	2.00	1	4710.71	16.00	120.00	190.60	89.90
94	3.00	4	2.00	1	4443.85	16.00	150.00	191.00	80.90
95	3.00	4	2.00	1	5140.32	17.00	150.00	194.30	103.30
98	3.00	5	2.00	1	3981.36	16.00	120.00	178.00	70.30
99	3.00	5	2.00	1	3823.42	16.00	120.00	182.00	63.60
100	3.00	4	2.00	1	5264.64	21.00	180.00	202.00	106.60
101	2.00	11	1.00	1	5388.77	19.00	150.00	187.00	115.20
102	2.00	11	1.00	1	3436.71	17.00	180.00	163.00	58.20
103	2.00	11	1.00	1	3724.69	17.00	180.00	170.00	65.20
104	2.00	11	1.00	1	4335.01	17.00	180.00	180.00	81.80
105	2.00	11	1.00	1	4638.22	17.00	180.00	184.00	90.40
106	2.00	11	1.00	1	4041.03	18.00	180.00	184.50	70.90
107	2.00	11	1.00	1	4080.45	16.00	180.00	175.50	74.50
111	2.00	11	1.00	1	4029.65	18.00	180.00	181.00	71.80
112	2.00	11	1.00	1	3055.65	14.00	180.00	152.50	47.90
113	2.00	11	1.00	1	3719.61	16.00	180.00	169.00	64.90
114	2.00	11	1.00	1	4028.68	14.00	180.00	181.50	69.60
115	2.00	11	1.00	1	3677.10	16.00	180.00	175.00	61.30
123	3.00	6	2.00	1	4352.02	21.00	240.00	190.00	80.70
124	3.00	6	2.00	1	4401.87	22.00	240.00	180.00	86.50
125	3.00	5	2.00	1	4023.62	18.00	180.00	181.00	71.60
126	3.00	5	2.00	1	3782.39	19.00	180.00	175.50	66.10
129	3.00	4	2.00	1	4604.67	19.00	180.00	196.00	85.90
130	3.00	4	2.00	1	4758.38	19.00	180.00	196.00	91.00
131	3.00	8	1.00	2	2548.67	17.00	90.00	163.00	65.70
132	3.00	8	1.00	1	3167.49	15.00	90.00	158.00	50.10
133	3.00	8	1.00	1	4647.81	22.00	90.00	184.00	93.20
134	2.00	14	2.00	1	4632.06	20.00	210.00	193.00	88.40
135	2.00	14	2.00	1	4302.50	16.00	240.00	185.00	78.40
136	2.00	14	2.00	1	3939.39	18.00	300.00	178.00	69.90
137	2.00	14	2.00	1	4153.95	15.00	240.00	180.00	74.80
138	2.00	14	2.00	1	4703.58	16.00	135.00	190.50	89.70
139	2.00	14	2.00	1	4870.60	24.00	240.00	193.00	98.30
140	2.00	14	2.00	2	2738.70	14.00	180.00	173.00	74.00

141	3.00	8	1.00	1	4544.54	19.00	120.00	193.00	85.00
142	3.00	4	2.00	1	4830.61	18.00	180.00	196.00	92.90
143	3.00	4	2.00	1	4939.90	17.00	120.00	198.00	95.30
144	3.00	4	2.00	1	4459.66	20.00	120.00	185.00	85.60
145	2.00	14	2.00	1	4076.23	14.00	240.00	185.00	69.90
146	2.00	14	2.00	1	3666.74	15.00	120.00	179.00	59.00
147	2.00	14	2.00	1	3758.41	14.00	120.00	172.00	64.10
148	2.00	14	2.00	1	3749.06	14.00	180.00	178.00	61.60
149	2.00	14	2.00	1	3501.17	14.00	240.00	173.00	55.20
150	2.00	14	2.00	1	3924.62	14.00	180.00	183.00	65.60
151	2.00	14	2.00	2	2567.51	15.00	90.00	165.00	65.50
152	2.00	14	2.00	2	2096.68	15.00	90.00	153.00	38.90
153	2.00	14	2.00	1	4323.18	15.00	135.00	191.00	76.40
154	2.00	14	2.00	2	2528.34	15.00	135.00	165.00	63.10
155	2.00	14	2.00	2	2358.41	15.00	120.00	166.00	52.50
156	2.00	14	2.00	2	2271.10	15.00	120.00	162.00	47.90
159	3.00	6	2.00	1	5099.40	23.00	240.00	196.00	104.30
160	2.00	14	2.00	2	2233.97	15.00	120.00	160.00	46.00
184	3.00	5	2.00	2	2443.92	20.00	120.00	167.00	60.00
190	3.00	5	2.00	1	4102.30	27.00	240.00	184.50	77.40
192	3.00	6	2.00	1	4513.56	25.00	240.00	183.00	90.60
211	3.00	7	1.00	1	4974.62	16.00	120.00	185.00	100.70
212	3.00	7	1.00	1	3451.05	16.00	90.00	175.00	53.80
213	3.00	7	1.00	1	3438.14	15.00	120.00	170.00	54.70
214	3.00	7	1.00	1	3371.08	15.00	90.00	165.00	54.30
217	2.00	14	2.00	1	4768.21	18.00	240.00	198.00	90.10
218	2.00	14	2.00	1	3993.48	18.00	240.00	181.00	70.60
219	3.00	5	2.00	1	4007.50	18.00	150.00	182.00	70.70
220	3.00	5	2.00	2	2331.45	16.00	150.00	163.00	51.90
224	2.00	14	2.00	2	2468.03	19.00	240.00	168.00	60.80
225	2.00	14	2.00	2	2359.19	16.00	240.00	163.00	53.60
226	2.00	14	2.00	1	3859.90	16.00	240.00	176.00	67.00
227	2.00	14	2.00	1	4059.79	18.00	240.00	181.00	72.80
228	2.00	14	2.00	1	4580.55	19.00	240.00	196.00	85.10
229	2.00	14	2.00	1	4454.05	17.00	240.00	190.00	82.10
230	2.00	14	2.00	1	4656.39	22.00	240.00	193.00	90.20
231	2.00	10	2.00	2	2568.53	21.00	270.00	173.00	67.00
232	2.00	10	2.00	2	2403.09	21.00	200.00	168.00	57.80
233	2.00	10	2.00	2	2506.65	17.00	180.00	169.00	62.00
234	2.00	10	2.00	2	2730.03	17.00	120.00	178.00	74.00
235	2.00	10	2.00	2	2703.88	24.00	270.00	176.00	76.20
236	2.00	10	2.00	2	2472.28	23.00	270.00	174.50	61.80
237	2.00	11	1.00	1	4666.24	17.00	120.00	186.00	90.60
238	3.00	7	1.00	1	3549.77	16.00	90.00	170.00	58.90
240	2.00	11	1.00	1	4378.84	14.00	180.00	181.00	81.40
241	3.00	7	1.00	1	3559.56	16.00	210.00	175.00	57.40
242	3.00	7	1.00	1	3123.30	16.00	120.00	160.00	48.40

243	3.00	5	2.00	1	4170.03	16.00	120.00	182.00	75.10
244	2.00	13	1.00	2	2338.15	17.00	120.00	155.00	54.30
245	2.00	13	1.00	1	2648.69	14.00	90.00	144.00	37.50
246	2.00	13	1.00	2	2390.06	14.00	130.00	162.00	54.70
247	2.00	13	1.00	2	2445.45	17.00	90.00	173.00	57.50
248	2.00	13	1.00	1	3307.74	16.00	120.00	168.00	51.60
249	2.00	13	1.00	2	2494.27	15.00	90.00	172.00	59.70
251	2.00	11	1.00	1	4018.15	27.00	180.00	170.00	79.90
254	2.00	11	1.00	1	4141.08	24.00	180.00	176.00	80.30
255	2.00	11	1.00	1	4536.09	23.00	180.00	190.00	87.80
256	2.00	11	1.00	1	4333.87	23.00	180.00	176.00	86.20
257	2.00	11	1.00	1	4136.79	22.00	180.00	177.00	78.80
258	2.00	11	1.00	1	3792.67	24.00	180.00	172.00	70.20
259	2.00	11	1.00	1	4415.84	23.00	120.00	184.00	86.00
263	2.00	13	1.00	1	3724.53	17.00	120.00	173.00	64.10
264	2.00	13	1.00	2	2344.33	21.00	180.00	168.00	54.20
265	2.00	13	1.00	1	2822.40	15.00	180.00	148.00	42.30
266	2.00	13	1.00	1	4979.74	16.00	120.00	183.00	101.60
270	2.00	13	1.00	2	2259.40	29.00	90.00	159.00	54.60
271	3.00	5	2.00	1	4268.33	15.00	120.00	183.00	77.50
273	3.00	4	2.00	1	4401.21	16.00	150.00	180.00	83.50
274	3.00	4	2.00	1	4397.05	18.00	150.00	187.00	81.80
290	2.00	14	2.00	1	4074.55	18.00	300.00	187.00	71.10
291	2.00	14	2.00	1	3949.26	16.00	240.00	177.00	69.60
292	2.00	14	2.00	1	4486.79	16.00	240.00	196.00	80.50
293	2.00	14	2.00	2	2534.67	15.00	180.00	174.00	61.80
295	3.00	5	2.00	2	2388.36	16.00	120.00	164.00	55.20
297	3.00	6	2.00	1	4292.20	21.00	130.00	181.00	82.00
299	2.00	14	2.00	2	2182.15	27.00	240.00	160.00	48.70
300	2.00	14	2.00	1	3808.46	17.00	150.00	182.00	63.60
301	2.00	10	2.00	2	2386.29	23.00	180.00	172.00	57.00
306	2.00	14	2.00	1	4223.49	17.00	90.00	180.00	78.10
307	2.00	14	2.00	1	4310.55	18.00	120.00	189.00	78.20
308	3.00	4	2.00	1	4735.70	17.00	120.00	203.00	86.70
309	3.00	4	2.00	1	4895.59	17.00	120.00	200.00	93.10
313	3.00	4	2.00	1	4423.01	17.00	240.00	188.00	81.80
314	3.00	4	2.00	1	4428.14	17.00	240.00	186.00	82.70
315	3.00	4	2.00	1	3899.57	15.00	150.00	184.00	64.90
316	3.00	4	2.00	1	4568.63	16.00	240.00	187.00	86.50
317	3.00	4	2.00	1	4415.82	16.00	240.00	189.00	80.70
322	3.00	4	2.00	2	2736.69	25.00	180.00	176.00	78.70
323	3.00	4	2.00	2	2507.30	22.00	180.00	170.00	64.30
324	3.00	4	2.00	2	2648.29	24.00	210.00	184.50	71.20
325	2.00	14	2.00	1	4142.95	15.00	300.00	179.00	74.80
326	2.00	14	2.00	1	4531.54	20.00	240.00	194.00	84.70
327	2.00	14	2.00	2	2455.68	16.00	180.00	170.00	58.20
328	2.00	14	2.00	2	2408.19	21.00	210.00	167.00	58.30

329	2.00	14	2.00	2	2454.12	14.00	300.00	176.00	56.00
330	2.00	14	2.00	1	4150.63	15.00	180.00	186.00	72.50

total_body	total_body	intralasteli	ekstralaste	edemos_ir	Lieknoji_kı	Lieknoji_kı	Raumenu_	Raumenu_	Kaires_ran
39.20	59.00	25.60	13.60	0.35	54.40	81.80	50.60	76.10	3.54
53.90	61.80	35.10	18.80	0.35	74.90	85.80	69.80	80.00	4.82
47.60	52.80	31.40	16.20	0.34	66.10	73.40	60.90	67.50	4.12
48.00	59.80	31.40	16.60	0.35	66.70	83.00	62.00	77.20	4.22
47.40	58.70	31.00	16.40	0.35	65.90	81.60	61.20	75.80	4.06
49.80	61.70	32.40	17.40	0.35	69.10	85.60	64.40	79.80	4.35
44.00	58.90	28.90	15.10	0.34	61.10	81.70	56.80	76.00	3.85
45.40	61.40	29.60	15.80	0.35	63.10	85.40	58.70	79.40	3.97
46.90	61.30	30.70	16.20	0.35	65.10	85.10	60.70	79.30	4.05
43.50	59.10	28.40	15.10	0.35	60.40	82.00	56.10	76.20	3.84
42.60	59.30	27.90	14.70	0.35	59.20	82.30	55.00	76.50	3.66
65.40	65.40	42.20	23.20	0.35	90.90	90.90	84.90	84.90	6.01
31.30	54.60	20.30	11.00	0.35	43.50	76.00	40.20	70.20	2.60
31.50	52.20	20.70	10.80	0.34	43.80	72.60	40.30	66.80	2.59
34.50	56.70	22.40	12.10	0.35	47.90	78.80	44.40	73.00	2.93
28.70	55.50	18.60	10.10	0.35	39.90	77.20	36.90	71.40	2.32
32.20	54.70	21.00	11.20	0.35	44.70	75.90	41.30	70.10	2.73
42.10	61.50	27.60	14.50	0.34	58.50	85.40	54.50	79.60	3.75
39.00	63.20	25.60	13.40	0.34	54.20	87.80	50.50	81.80	3.41
39.20	60.70	25.70	13.50	0.34	54.50	84.40	50.70	78.50	3.37
43.30	60.10	28.50	14.80	0.34	60.10	83.40	55.90	77.60	3.70
46.20	58.40	30.30	15.90	0.34	64.20	81.10	59.60	75.30	3.96
41.20	61.50	26.90	14.30	0.35	57.20	85.40	53.30	79.60	3.57
40.40	55.20	26.50	13.90	0.34	56.10	76.60	51.90	70.90	3.42
41.40	64.00	26.70	14.70	0.36	57.50	88.90	53.70	83.00	3.54
30.00	67.00	19.30	10.70	0.36	41.70	93.10	39.00	87.10	2.58
40.70	62.30	26.30	14.40	0.35	56.50	86.40	52.70	80.60	3.53
39.20	65.80	25.50	13.70	0.35	54.50	91.60	50.90	85.50	3.40
35.60	56.50	23.20	12.40	0.35	49.40	78.40	45.80	72.70	3.03
34.80	56.68	22.60	12.20	0.35	48.40	78.83	44.80	72.96	2.97
38.50	62.60	24.90	13.60	0.35	53.50	87.10	49.90	81.20	3.32
40.20	63.20	26.00	14.20	0.35	55.80	87.70	52.10	81.90	3.52
32.00	66.40	20.60	11.40	0.36	44.50	92.30	41.60	86.30	2.76
36.30	64.02	23.40	12.90	0.36	50.40	88.89	47.10	83.07	3.15
48.20	56.70	31.20	17.00	0.35	67.00	78.90	62.00	73.00	4.19
41.50	55.30	27.30	14.20	0.34	57.70	77.00	53.30	71.10	3.60
47.70	57.30	31.80	15.90	0.33	66.20	79.50	61.40	73.70	4.14
51.90	55.20	34.00	17.90	0.34	72.10	76.60	66.70	70.90	4.41
34.80	54.60	22.60	12.20	0.35	48.40	75.90	44.70	70.10	2.99
35.90	52.20	23.30	12.60	0.35	49.90	72.50	45.90	66.70	3.01
35.20	51.80	23.20	12.00	0.34	48.90	71.90	45.00	66.20	3.00
35.40	51.10	23.10	12.30	0.35	49.20	71.00	45.20	65.20	2.90
34.80	58.40	22.80	12.00	0.35	48.40	81.20	44.90	75.30	2.95
42.20	58.50	27.90	14.30	0.34	58.60	81.20	54.40	75.40	3.65
39.00	62.30	25.70	13.30	0.34	54.10	86.30	50.50	80.60	3.44
40.30	63.60	26.40	13.90	0.35	56.00	88.30	52.30	82.50	3.64

39.10	66.20	25.10	14.00	0.36	54.30	91.90	50.80	86.00	3.37
29.30	66.60	19.00	10.30	0.35	40.70	92.50	38.10	86.60	2.52
47.40	57.90	30.70	16.70	0.35	63.70	78.90	59.10	73.20	4.06
34.90	61.00	23.30	11.60	0.33	48.50	84.90	45.10	78.90	2.99
30.70	59.40	20.00	10.70	0.35	42.70	82.60	39.60	76.60	2.57
31.00	55.10	20.40	10.60	0.34	43.00	76.40	39.80	70.70	2.67
34.80	57.70	22.30	12.50	0.36	48.30	80.10	44.80	74.30	2.91
43.10	59.30	28.10	15.00	0.35	59.80	82.30	55.60	76.50	3.82
43.60	59.80	28.50	15.10	0.35	60.50	83.00	56.30	77.20	3.94
45.60	60.10	29.70	15.90	0.35	63.40	83.50	58.90	77.60	3.95
37.90	59.20	24.40	13.50	0.36	52.60	82.20	48.90	76.40	3.24
53.70	57.50	35.10	18.60	0.35	74.60	79.90	69.20	74.10	4.81
50.60	57.00	33.10	17.50	0.35	70.30	79.20	65.10	73.30	4.43
50.00	55.60	32.80	17.20	0.34	69.40	77.20	64.20	71.40	4.36
48.60	60.00	31.80	16.80	0.35	67.50	83.40	62.80	77.60	4.33
55.00	53.00	36.30	18.70	0.34	76.40	73.70	70.40	67.90	4.82
41.50	59.10	27.10	14.40	0.35	57.70	82.10	53.60	76.30	3.65
41.30	65.00	26.90	14.40	0.35	57.30	90.10	53.60	84.30	3.49
56.30	52.90	37.20	19.10	0.34	78.20	73.40	72.10	67.70	4.91
65.20	56.60	42.40	22.80	0.35	90.50	78.56	83.90	72.83	5.83
36.60	62.90	23.60	13.00	0.36	50.80	87.30	47.40	81.50	3.26
39.60	60.80	25.70	13.90	0.35	55.00	84.40	51.20	78.60	3.53
47.20	57.80	30.60	16.60	0.35	65.50	80.10	60.80	74.40	4.15
49.50	54.70	32.20	17.30	0.35	68.80	76.10	63.60	70.30	4.29
43.60	61.50	28.40	15.20	0.35	60.60	85.50	56.40	79.60	3.79
43.40	58.20	28.30	15.10	0.35	60.30	80.90	56.00	75.10	3.78
44.10	61.40	28.60	15.50	0.35	61.20	85.20	57.00	79.40	3.90
30.70	64.00	19.90	10.80	0.35	42.70	89.10	39.80	83.00	2.75
40.10	61.70	25.90	14.20	0.36	55.70	85.80	51.90	79.90	3.61
43.20	62.10	28.00	15.20	0.35	60.00	86.20	55.90	80.30	3.81
39.80	64.90	25.50	14.30	0.36	55.30	90.20	51.70	84.30	3.50
46.80	58.00	30.90	15.90	0.34	65.00	80.50	60.30	74.70	4.22
50.30	58.10	33.30	17.00	0.34	69.80	80.70	64.80	74.90	4.51
43.20	60.30	28.30	14.90	0.35	60.00	83.80	55.80	77.90	3.76
39.40	59.70	25.60	13.80	0.35	54.70	82.80	50.90	77.10	3.28
50.40	58.70	32.80	17.60	0.35	70.00	81.50	65.00	75.70	4.48
56.30	61.90	36.50	19.80	0.35	78.20	80.10	72.90	80.10	5.07
33.30	50.70	21.70	11.60	0.35	46.30	70.50	42.50	64.70	2.77
32.40	64.60	20.80	11.60	0.36	45.00	89.80	42.00	83.80	2.75
51.00	54.70	33.30	17.70	0.35	70.90	76.10	65.50	70.30	4.45
49.60	56.00	32.70	16.90	0.34	68.90	77.90	63.80	72.10	4.26
45.10	57.50	29.90	15.20	0.34	62.70	80.00	58.10	74.10	3.84
39.40	56.40	26.40	13.00	0.33	54.70	78.30	50.70	72.60	3.29
44.90	60.10	29.20	15.70	0.35	62.30	83.30	58.00	77.60	4.00
50.30	56.00	33.00	17.30	0.34	69.90	77.90	64.70	72.10	4.36
56.40	57.40	37.20	19.20	0.34	78.40	79.80	72.70	74.00	4.99
39.30	53.10	25.70	13.60	0.35	54.60	73.80	50.30	68.00	3.25

49.80	58.60	32.30	17.50	0.35	69.10	81.30	64.20	75.50	4.28
51.40	55.40	33.70	17.70	0.35	71.40	76.90	66.00	71.10	4.39
54.40	57.00	35.90	18.50	0.34	75.60	79.30	70.00	73.40	4.74
52.10	60.90	34.10	18.00	0.35	72.30	84.50	67.30	78.70	4.54
43.10	61.80	28.10	15.00	0.35	59.90	85.70	55.80	79.80	3.72
38.20	64.80	25.00	13.20	0.35	53.10	90.00	49.60	84.10	3.25
38.70	60.40	25.20	13.50	0.35	53.70	83.80	50.00	78.00	3.37
39.10	63.50	25.10	14.00	0.36	54.30	88.10	50.70	82.30	3.39
36.40	66.00	23.30	13.10	0.36	50.50	91.50	47.30	85.70	3.12
42.60	64.90	27.60	15.00	0.35	59.10	90.10	55.30	84.30	3.76
34.60	52.90	22.40	12.20	0.35	48.00	73.30	44.30	67.70	2.95
23.90	61.40	15.30	8.60	0.36	33.20	85.30	30.90	79.40	2.02
46.30	60.60	29.80	16.50	0.36	64.30	84.20	59.80	78.30	4.02
34.10	54.10	21.80	12.30	0.36	47.30	69.30	43.70	69.30	2.80
30.80	58.60	19.70	11.10	0.36	42.80	81.50	39.70	75.60	2.52
29.30	61.20	18.80	10.50	0.36	40.70	85.00	37.90	79.20	2.45
58.20	55.60	38.20	20.00	0.34	80.80	77.50	74.80	71.70	5.10
28.20	60.30	18.20	10.00	0.35	39.20	83.90	36.40	77.90	2.33
34.30	57.20	21.70	12.60	0.37	47.70	79.50	44.20	73.70	2.88
45.20	58.40	29.40	15.80	0.35	62.80	81.10	58.30	75.30	3.87
55.20	60.90	35.80	19.40	0.35	76.60	84.50	71.40	78.80	4.89
51.70	51.30	34.00	17.70	0.34	71.80	71.30	66.10	65.60	4.41
36.60	68.10	23.50	13.10	0.36	50.90	94.60	47.70	88.70	3.17
34.60	63.30	22.70	11.90	0.34	48.00	87.80	44.80	81.90	2.91
34.80	64.00	22.60	12.20	0.35	48.40	89.10	45.20	83.20	2.96
53.70	59.60	35.00	18.70	0.35	74.60	82.80	69.30	76.90	4.77
42.80	60.60	28.00	14.80	0.35	59.40	84.10	55.30	78.30	3.69
42.00	59.40	27.50	14.50	0.35	58.40	82.60	54.20	76.70	3.59
28.70	55.30	18.90	9.80	0.34	39.80	76.70	36.90	71.10	2.38
33.20	54.60	21.50	11.70	0.35	46.10	75.80	42.60	70.10	2.76
30.30	56.50	19.70	10.60	0.35	42.10	78.50	39.00	72.80	2.51
41.20	61.50	26.90	14.30	0.35	57.20	85.40	53.30	79.60	3.61
43.90	60.30	28.60	15.30	0.35	61.00	83.80	56.70	77.90	3.81
52.20	61.30	33.80	18.40	0.35	72.50	85.20	67.50	79.30	4.65
48.60	59.20	32.00	16.60	0.34	67.50	82.20	62.70	76.40	4.29
53.50	59.30	34.70	18.80	0.35	74.30	82.40	69.10	76.60	4.73
36.20	54.00	23.50	12.70	0.35	50.30	75.10	46.40	69.30	2.97
32.40	56.10	21.20	11.20	0.35	45.00	77.90	41.71	72.20	2.67
33.00	53.20	21.50	11.50	0.35	45.80	73.90	42.30	68.20	2.70
38.80	52.40	25.30	13.50	0.35	53.90	72.80	49.60	67.00	3.18
40.10	52.60	25.60	14.50	0.36	55.70	73.10	51.30	67.30	3.27
36.90	59.70	23.50	13.40	0.36	51.20	82.80	47.60	77.00	3.09
51.80	57.20	33.60	18.20	0.35	72.00	79.50	66.70	73.60	4.59
37.00	62.80	24.10	12.90	0.35	51.40	87.30	47.90	81.30	3.27
46.60	57.20	30.50	16.10	0.35	64.70	79.50	60.00	73.70	4.12
36.90	64.30	24.10	12.80	0.35	51.20	89.20	47.90	83.40	3.12
32.20	66.50	20.70	11.50	0.36	44.70	92.40	41.80	86.40	2.77

43.20	57.50	28.60	14.60	0.34	60.00	79.90	55.60	74.00	3.70
28.90	53.20	18.60	10.30	0.36	40.20	74.00	37.00	68.10	2.47
25.00	66.60	15.90	9.10	0.36	34.70	92.50	32.50	86.60	2.13
30.40	55.50	19.40	11.00	0.36	42.20	77.10	39.10	71.40	2.60
33.90	59.00	21.60	12.30	0.36	47.10	81.90	43.70	76.00	2.82
33.30	64.50	21.30	12.00	0.36	46.20	89.50	43.20	83.70	2.85
33.70	56.50	21.70	12.00	0.36	46.80	78.40	43.40	72.70	2.90
45.90	57.40	29.90	16.00	0.35	63.70	79.70	59.10	73.90	4.08
45.40	56.60	29.70	15.70	0.35	63.00	78.50	58.40	72.80	4.05
53.60	61.10	34.70	18.90	0.35	74.50	84.90	69.30	79.00	4.70
52.20	60.60	33.90	18.30	0.35	72.50	84.10	67.50	78.30	4.65
47.40	60.20	30.90	16.50	0.35	65.90	83.60	61.30	77.80	4.30
42.00	59.80	27.10	14.90	0.35	58.30	83.00	54.20	77.20	3.73
53.10	61.80	34.40	18.70	0.35	73.70	85.70	68.70	79.90	4.72
39.00	60.90	25.50	13.50	0.35	54.20	84.60	50.40	78.70	3.41
31.20	57.80	20.20	11.00	0.35	43.30	79.90	40.20	74.20	2.64
27.10	64.10	17.50	9.60	0.35	37.70	89.10	35.20	83.20	2.26
51.00	50.20	33.00	18.00	0.35	70.90	69.80	65.10	64.10	4.32
30.20	55.20	19.50	10.70	0.35	42.00	76.90	38.80	71.00	2.56
44.10	56.90	28.90	15.20	0.35	61.20	78.97	56.70	73.16	3.72
47.10	56.40	30.70	16.40	0.35	65.40	78.30	60.60	72.60	4.17
47.20	57.80	30.60	16.60	0.35	65.50	80.10	60.80	74.40	3.95
43.00	60.50	28.20	14.80	0.35	59.70	84.00	55.60	78.20	3.71
41.60	59.80	27.20	14.60	0.35	58.10	83.50	54.00	77.60	3.68
49.20	61.10	32.20	17.00	0.35	68.40	85.00	63.70	79.10	4.26
35.40	57.30	23.00	12.40	0.35	49.10	79.40	45.60	73.80	2.94
30.10	54.50	19.60	10.50	0.35	41.80	75.70	38.60	69.90	2.40
51.40	62.70	34.00	17.40	0.34	71.40	87.10	66.60	81.20	4.55
39.00	61.30	25.70	13.30	0.34	39.70	81.50	36.90	75.80	3.25
31.00	54.40	20.10	10.90	0.35	54.20	85.20	50.50	79.40	2.52
36.80	55.10	23.70	13.10	0.36	43.10	75.60	39.80	69.80	2.99
44.40	56.90	29.30	15.10	0.34	61.60	78.90	57.10	73.10	3.80
46.30	59.20	30.30	16.00	0.35	64.30	82.20	59.80	76.50	4.02
51.40	59.30	33.50	17.90	0.35	71.40	82.40	66.30	76.50	4.33
53.20	57.10	34.80	18.40	0.35	73.90	79.40	68.50	73.60	4.56
48.50	59.30	31.40	17.10	0.35	67.30	82.30	62.60	76.50	4.12
48.40	58.50	31.70	16.70	0.35	67.20	81.30	62.40	75.50	4.30
41.00	63.20	26.40	14.60	0.36	56.90	87.70	53.10	81.80	3.54
49.50	57.20	31.60	17.90	0.36	68.70	79.40	63.70	73.60	4.13
48.00	59.50	31.40	16.60	0.35	66.70	82.70	62.00	76.80	4.25
41.00	52.10	26.20	14.80	0.36	56.90	72.30	52.40	66.60	3.39
35.90	55.80	22.90	13.00	0.36	49.90	77.60	46.20	71.90	2.95
41.00	57.60	26.00	15.00	0.37	57.00	80.10	52.80	74.20	3.42
43.60	58.30	28.20	15.40	0.35	60.60	81.00	56.20	75.10	3.81
48.30	57.00	31.20	17.10	0.35	67.10	79.20	62.20	73.40	4.15
33.40	57.40	21.30	12.10	0.36	46.40	79.70	43.00	73.90	2.76
32.30	55.40	20.60	11.70	0.36	44.90	77.00	41.50	71.20	2.73

34.70	62.00	22.10	12.60	0.36	48.20	86.10	44.90	80.20	2.85
45.10	62.20	29.10	16.00	0.35	62.60	86.30	58.40	80.60	3.88

Desines_ra	Kaires_koj	Desines_kc	Liemens_ra	RRMI_raur	FFMI_adj	Baltymu_k	Baltymu_k	Mineralu_l	Mineralu_l
3.56	9.18	9.20	25.12	4.20	21.42	11.40	17.10	3.80	5.70
4.77	13.07	12.83	34.32	5.60	20.70	15.90	18.20	5.10	5.80
4.11	10.98	11.23	30.46	2.50	20.12	13.30	14.80	5.20	5.80
4.26	11.52	11.36	30.64	4.50	19.46	14.00	17.40	4.70	5.80
4.05	11.23	11.31	30.55	4.10	18.55	13.80	17.10	4.70	5.80
4.26	11.95	11.77	32.07	5.60	18.53	14.60	18.10	4.70	5.80
3.86	10.33	10.34	28.41	4.10	18.86	12.80	17.10	4.30	5.70
4.00	10.79	10.86	29.09	5.40	18.93	13.30	18.00	4.40	6.00
4.06	11.10	11.16	30.34	5.30	17.42	13.80	18.00	4.40	5.80
3.85	10.18	10.18	28.05	4.20	18.38	12.60	17.10	4.30	5.80
3.58	10.17	9.92	27.68	4.30	18.01	12.40	17.20	4.20	5.80
5.92	16.36	16.01	40.60	9.30	25.91	19.50	19.50	6.00	6.00
2.60	7.20	7.19	20.61	2.90	16.64	8.90	15.60	3.30	5.80
2.62	7.17	7.06	20.86	2.40	16.25	8.80	14.60	3.50	5.80
2.97	7.96	8.07	22.47	3.40	15.95	9.90	16.30	3.50	5.80
2.30	6.52	6.70	19.06	3.10	15.57	8.20	15.90	3.00	5.80
2.74	7.53	7.50	20.80	2.90	16.69	9.10	15.40	3.40	5.80
3.81	9.64	9.79	27.51	5.50	17.29	12.40	18.10	4.00	5.80
3.47	9.09	9.22	25.31	6.70	17.74	11.50	18.60	3.70	6.00
3.41	9.29	9.32	25.31	5.00	18.37	11.50	17.80	3.80	5.90
3.75	10.00	10.70	28.38	4.70	16.64	12.60	17.50	4.20	5.80
3.94	11.07	11.37	29.26	4.00	18.99	13.40	16.90	4.60	5.80
3.47	10.00	9.77	26.49	5.40	18.98	12.10	18.10	3.90	5.80
3.44	9.65	9.83	25.57	3.00	18.35	11.50	15.70	4.20	5.70
3.50	10.00	9.90	26.75	7.50	17.75	12.30	19.00	3.80	5.90
2.55	7.09	7.04	19.74	12.60	18.05	9.00	20.10	2.70	6.00
3.56	9.87	10.02	25.73	5.90	19.31	12.00	18.30	3.80	5.80
3.40	9.12	9.23	25.75	10.20	16.82	11.70	19.70	3.60	6.10
3.03	8.32	8.47	22.96	3.40	17.44	10.20	16.20	3.60	5.70
2.97	8.06	8.18	22.62	3.40	17.10	10.00	16.29	3.60	5.86
3.27	9.16	9.15	25.00	6.30	18.57	11.40	18.60	3.60	5.90
3.49	9.51	9.46	26.12	6.70	18.26	11.90	18.70	3.70	5.80
2.75	7.52	7.46	21.10	11.20	18.05	9.60	19.90	2.90	6.00
3.09	8.48	8.34	24.04	7.50	17.52	10.80	19.05	3.30	5.82
4.22	11.47	11.47	30.65	3.50	19.27	13.80	16.30	5.00	5.90
3.62	9.63	9.66	26.80	3.10	19.42	11.80	15.80	4.40	5.90
4.07	11.17	10.96	31.07	3.60	18.24	13.70	16.40	4.80	5.80
4.36	12.48	12.25	33.21	3.00	19.09	14.80	15.70	5.40	5.70
3.03	8.09	8.13	22.46	2.90	17.88	9.90	15.50	3.70	5.80
3.00	8.43	8.57	22.89	2.40	18.82	10.00	14.50	4.00	5.80
3.04	8.04	8.19	22.74	2.40	17.79	9.80	14.40	3.90	5.70
2.94	8.28	8.51	22.58	2.20	17.90	9.80	14.10	4.00	5.80
2.98	8.24	8.28	22.46	4.00	18.42	10.10	16.90	3.50	5.90
3.66	9.82	9.83	27.44	4.00	19.72	12.20	16.90	4.20	5.80
3.41	9.10	8.94	25.61	5.90	18.23	11.50	18.30	3.60	5.70
3.63	9.06	9.22	26.75	7.10	17.87	12.00	18.90	3.70	5.80

3.36	9.42	9.36	25.29	10.60	19.40	11.70	19.80	3.50	5.90
2.52	6.79	6.84	19.43	11.50	18.05	8.80	20.00	2.60	5.90
4.09	11.45	11.51	29.98	3.50	19.47	13.20	16.40	4.60	5.70
2.99	7.90	7.98	23.24	5.20	17.39	10.20	17.90	3.40	6.00
2.53	7.16	7.05	20.29	4.40	16.35	8.90	17.20	3.10	6.00
2.67	7.07	7.01	20.38	3.00	16.58	8.80	15.60	3.20	5.70
2.89	8.18	8.14	22.67	3.70	16.32	10.00	16.60	3.50	5.80
3.83	9.81	9.97	28.16	4.30	18.46	12.50	17.20	4.20	5.80
3.94	10.28	10.20	27.94	4.50	19.22	12.70	17.40	4.20	5.80
3.99	11.04	11.12	28.80	4.70	18.75	13.30	17.50	4.50	5.90
3.22	9.46	9.43	23.55	4.30	19.23	11.00	17.20	3.70	5.80
4.84	12.44	12.62	34.48	3.70	19.92	15.50	16.60	5.40	5.80
4.47	11.72	11.78	32.70	3.50	17.98	14.50	16.30	5.20	5.90
4.36	11.58	11.63	32.26	3.10	18.46	14.20	15.80	5.20	5.80
4.33	11.30	11.32	31.51	4.70	17.83	14.20	17.60	4.70	5.80
4.77	13.15	12.94	34.73	2.60	19.36	15.40	14.90	6.00	5.80
3.68	9.51	9.62	27.14	4.30	18.33	12.10	17.20	4.10	5.80
3.48	9.91	9.88	26.84	8.50	17.18	12.30	19.30	3.70	5.80
5.04	12.93	13.29	35.94	2.50	17.82	15.80	14.80	6.10	5.70
5.80	15.79	16.14	40.34	3.40	25.45	18.70	16.23	6.60	5.73
3.29	8.83	8.65	23.38	6.40	20.16	10.80	18.60	3.40	5.80
3.55	9.30	9.40	25.42	5.00	19.64	11.60	17.80	3.80	5.80
4.12	11.35	11.28	29.89	3.70	20.22	13.60	16.60	4.70	5.70
4.22	11.89	11.89	31.32	2.90	20.08	14.10	15.60	5.20	5.80
3.77	9.96	10.22	28.66	5.50	17.53	12.80	18.10	4.20	5.90
3.74	10.54	10.40	27.55	3.90	19.85	12.60	16.90	4.30	5.80
3.92	10.35	10.42	28.41	5.40	18.62	12.90	18.00	4.20	5.80
2.76	7.19	7.11	19.99	7.70	20.04	9.10	19.00	2.90	6.10
3.60	9.59	9.55	25.55	5.60	20.17	11.80	18.20	3.80	5.90
3.80	9.97	10.17	28.15	5.80	18.12	12.70	18.20	4.10	5.90
3.46	9.49	9.39	25.86	8.60	18.36	11.90	19.40	3.60	5.90
4.24	10.46	10.47	30.91	3.80	17.40	13.50	16.70	4.70	5.80
4.58	11.89	11.97	31.85	3.90	21.54	14.50	16.80	5.00	5.80
3.79	10.23	10.42	27.60	4.80	18.25	12.60	17.60	4.20	5.90
3.30	9.35	9.41	25.57	4.50	18.03	11.50	17.40	3.80	5.70
4.50	11.52	11.56	32.95	4.10	17.25	14.60	17.00	5.00	5.80
5.11	13.28	13.45	35.99	5.70	19.38	16.60	18.20	5.30	5.80
2.77	7.77	7.84	21.36	2.20	18.46	9.20	14.00	3.80	5.80
2.72	7.96	7.85	20.71	8.20	19.37	9.60	19.20	3.00	6.00
4.44	12.28	12.03	32.30	2.90	20.70	14.50	15.60	5.40	5.80
4.30	11.63	11.47	32.14	3.30	17.70	14.20	16.10	5.10	5.80
3.77	10.56	10.43	29.49	3.70	18.01	13.00	16.60	4.60	5.90
3.22	9.08	8.87	26.24	3.30	17.39	11.30	16.20	4.00	5.70
4.03	10.66	10.69	28.61	4.60	19.23	13.10	17.50	4.30	5.70
4.43	11.80	11.94	42.17	3.30	18.62	14.40	16.10	5.20	5.80
5.02	13.37	13.50	35.83	3.70	20.25	16.30	16.60	5.70	5.80
3.25	9.51	9.57	24.71	2.60	18.67	11.00	14.90	4.30	5.80

4.19	12.30	11.98	31.44	4.00	17.76	14.40	16.90	4.90	5.80
4.40	12.01	12.06	33.14	3.10	17.61	14.60	15.70	5.40	5.80
4.76	12.83	12.94	34.73	3.60	18.19	15.60	16.40	5.60	5.90
4.49	12.66	12.45	33.16	5.10	20.82	15.20	17.80	5.00	5.80
3.63	10.09	9.89	28.47	5.60	17.20	12.70	18.20	4.10	5.90
3.23	8.76	9.00	25.37	8.40	16.63	11.40	19.30	3.50	5.90
3.33	9.08	8.92	25.31	4.80	18.64	11.30	17.60	3.70	5.80
3.38	8.99	9.14	25.80	6.90	17.26	11.60	18.80	3.60	5.80
3.16	8.54	8.64	23.85	10.10	17.30	10.90	19.70	3.20	5.80
3.78	10.12	10.05	27.60	8.50	17.46	12.70	19.40	3.80	5.80
2.99	8.00	8.22	22.14	2.50	18.55	9.70	14.80	3.70	5.60
2.06	5.56	5.44	15.82	5.40	15.83	7.00	18.00	2.30	5.90
4.02	10.75	10.84	30.17	4.90	16.95	13.50	17.70	4.50	5.90
2.76	8.16	8.12	21.86	2.80	18.29	9.60	15.20	3.60	5.70
2.48	7.20	7.19	20.32	4.10	16.39	8.90	17.00	3.10	5.90
2.45	6.91	6.84	19.25	5.30	16.61	8.60	18.00	2.80	5.80
4.97	13.92	13.57	37.23	3.20	20.06	16.60	15.90	6.00	5.80
2.33	6.62	6.68	18.44	4.90	16.53	8.20	17.60	2.80	6.00
2.83	8.55	8.53	21.41	3.60	17.90	9.90	16.50	3.50	5.80
3.79	10.58	11.02	29.05	4.00	18.17	13.10	16.90	4.50	5.80
4.82	13.25	13.62	34.82	5.10	22.69	16.20	17.90	5.20	5.70
4.43	12.36	12.36	32.54	2.30	20.67	14.40	14.30	5.70	5.70
3.11	8.56	8.47	24.40	16.40	16.93	11.10	20.60	3.20	5.90
2.88	7.99	7.96	23.07	6.70	17.22	10.20	18.60	3.20	5.90
2.97	8.32	8.29	22.60	7.70	18.69	10.40	19.20	3.20	5.90
4.78	12.75	12.66	34.35	4.50	17.93	15.60	17.30	5.30	5.90
3.71	10.06	10.15	27.69	4.90	18.07	12.50	17.70	4.10	5.80
3.53	9.87	9.72	27.49	4.40	17.51	12.20	17.30	4.20	5.90
2.38	6.48	6.53	19.12	3.00	16.02	8.20	15.80	2.90	5.60
2.75	7.70	7.65	21.70	2.90	17.07	9.40	15.50	3.50	5.80
2.54	7.15	6.99	19.81	3.40	16.88	8.70	16.20	3.10	5.80
3.60	9.69	9.82	26.58	5.40	18.71	12.10	18.10	3.90	5.80
3.75	10.54	10.43	28.17	4.80	18.56	12.80	17.60	4.30	5.90
4.68	12.19	12.40	33.57	5.40	17.90	15.30	18.00	5.00	5.90
4.27	11.37	11.37	31.40	4.30	18.09	14.10	17.20	4.80	5.80
4.80	12.56	12.84	34.17	4.30	19.15	15.60	17.30	5.20	5.80
3.01	8.52	8.73	23.17	2.80	17.23	10.20	15.20	3.90	5.80
2.64	7.64	7.64	21.10	3.30	16.68	9.30	16.10	3.30	5.70
2.65	7.80	7.56	21.60	2.70	16.71	9.30	15.00	3.50	5.60
3.13	9.12	9.06	25.12	2.40	17.13	10.80	14.60	4.30	5.80
3.21	10.62	9.68	24.53	2.50	18.23	11.20	14.70	4.40	5.80
3.09	9.41	9.04	22.98	4.40	17.15	10.70	17.30	3.60	5.80
4.55	12.62	12.61	32.33	3.60	20.45	14.90	16.40	5.30	5.80
3.30	8.59	8.69	24.05	6.40	18.40	10.90	18.50	3.50	5.90
4.02	11.07	10.83	29.95	3.60	19.69	13.40	16.50	4.70	5.80
3.03	8.63	8.51	24.61	7.70	17.02	11.00	19.20	3.30	5.70
2.79	7.62	7.68	20.94	11.30	18.68	9.60	19.80	2.90	6.00

3.78	10.10	10.23	27.79	3.70	17.99	12.40	16.50	4.40	5.90
2.45	6.51	6.68	18.88	2.60	18.26	8.10	14.90	3.20	5.90
2.07	6.03	5.81	16.46	11.60	18.93	7.50	20.00	2.20	5.90
2.63	7.01	7.05	19.81	3.10	17.18	8.70	15.90	3.10	5.70
2.85	8.01	8.06	21.96	4.20	16.16	9.80	17.00	3.40	5.90
2.89	7.59	7.71	22.15	8.00	17.10	9.90	19.20	3.00	5.80
2.96	7.66	7.85	22.03	3.40	16.31	9.70	16.20	3.40	5.70
3.89	11.04	10.85	29.15	3.60	22.65	13.20	16.50	4.60	5.80
3.98	10.59	10.43	29.35	3.40	20.58	13.00	16.20	4.60	5.70
4.68	12.70	12.89	34.25	5.20	20.03	15.70	17.90	5.20	5.90
4.67	12.73	12.68	32.76	4.90	23.65	15.30	17.70	5.00	5.80
4.35	11.16	11.28	30.22	4.80	21.22	13.90	17.60	4.60	5.80
3.72	9.88	9.93	26.94	4.60	20.19	12.20	17.40	4.10	5.80
4.75	12.77	12.86	33.60	5.60	21.52	15.60	18.10	5.00	5.80
3.37	9.09	9.06	25.47	5.10	18.54	11.40	17.80	3.80	5.90
2.63	7.10	7.32	20.51	3.70	16.07	9.00	16.60	3.10	5.70
2.26	6.35	6.44	17.88	7.70	19.16	8.10	19.10	2.50	5.90
4.33	12.20	12.28	31.98	2.10	20.99	14.10	13.90	5.80	5.70
2.54	7.14	7.10	19.46	3.10	17.89	8.60	15.80	3.20	5.90
3.71	10.36	10.30	28.61	3.48	18.09	12.60	16.26	4.50	5.81
4.18	11.17	11.21	29.87	3.30	20.19	13.50	16.20	4.80	5.70
3.66	11.82	10.74	30.63	3.70	18.30	13.60	16.60	4.70	5.70
3.74	9.86	9.86	28.42	4.90	16.65	12.60	17.70	4.10	5.80
3.67	9.99	9.96	26.70	3.70	18.73	12.20	17.50	4.10	5.90
4.26	11.55	11.45	32.09	5.30	16.83	14.40	17.90	4.80	6.00
2.96	8.39	8.27	23.04	3.60	16.58	10.20	16.50	3.50	5.70
2.37	7.04	6.98	19.81	2.90	16.52	8.50	15.40	3.20	5.80
4.64	12.70	12.16	32.55	6.30	21.73	15.20	18.50	4.80	5.90
3.21	8.97	8.94	26.13	4.10	16.24	8.30	17.00	2.80	5.70
2.47	7.17	7.05	20.59	5.40	15.06	11.50	18.10	3.70	5.80
2.96	8.90	8.90	23.56	2.90	17.50	8.80	15.40	3.30	5.80
3.75	10.56	10.47	28.53	3.50	19.01	12.70	16.30	4.50	5.80
4.05	10.80	10.92	30.02	4.30	17.45	13.50	17.30	4.50	5.80
4.33	12.13	12.28	33.23	4.30	15.92	14.90	17.20	5.10	5.90
4.60	12.50	12.68	34.16	3.60	17.26	15.30	16.40	5.40	5.80
4.11	11.75	11.92	30.70	4.30	18.55	14.10	17.20	4.70	5.70
4.34	11.52	11.63	30.61	4.00	19.06	14.00	16.90	4.80	5.80
3.54	9.44	9.48	27.10	6.60	16.56	12.10	18.60	3.80	5.90
4.10	12.24	12.10	31.13	3.60	19.22	14.20	16.40	5.00	5.80
4.33	11.15	11.59	30.68	4.40	18.12	14.00	17.30	4.70	5.80
3.41	10.04	9.91	26.65	2.40	18.61	11.40	14.50	4.50	5.70
2.85	8.79	8.63	22.98	3.20	17.88	10.30	16.00	3.70	5.80
3.38	10.55	10.33	25.11	3.70	16.47	11.80	16.60	4.20	5.90
3.80	10.29	10.28	28.01	4.00	18.97	12.60	16.80	4.40	5.90
4.15	10.96	11.06	31.88	3.50	16.97	13.90	16.40	4.90	5.80
2.73	7.76	7.92	21.83	3.60	16.67	9.60	16.50	3.40	5.80
2.73	7.54	7.51	20.99	3.10	16.89	9.20	15.80	3.40	5.80

2.77	8.32	8.23	22.72	5.80	15.80	10.20	18.20	3.30	5.90
3.77	10.86	10.66	29.24	5.90	17.73	13.30	18.30	4.20	5.80

ENDOMOR	MEZOMOR	EKTOMORI	Riebalu_m	Riebalu_m	KMI_kuno_	Baltymai_r	Angliavanc	Angliavanc	Baltymu_ti
5.30	6.70	1.71	12.10	18.20	24.90	83.20	274.48	48.02	14.56
4.34	5.36	2.81	12.40	14.20	24.70	120.80	483.63	49.14	12.28
5.58	6.37	1.86	23.90	26.60	27.50	152.10	294.33	43.85	22.65
4.45	5.34	2.99	13.70	17.00	23.70	110.40	242.88	41.60	18.92
4.37	5.09	3.27	14.90	18.40	23.20	117.70	399.85	48.00	14.13
3.94	4.72	3.76	11.60	14.40	22.40	91.50	331.77	51.09	14.09
4.54	5.41	3.07	13.70	18.30	23.10	77.50	369.72	58.41	12.25
4.19	5.12	3.39	10.80	14.60	22.30	215.40	889.71	58.43	14.14
3.79	4.45	4.16	11.40	14.90	21.20	102.30	485.51	55.19	11.63
4.40	5.22	3.30	13.30	18.00	22.50	141.60	735.71	66.25	12.75
4.30	5.08	3.46	12.70	17.70	21.90	121.50	297.71	44.14	18.01
4.78	6.43	0.95	9.10	9.10	28.90	133.70	603.21	48.43	10.73
4.21	4.48	3.12	13.70	24.00	20.80	44.90	117.31	36.17	13.83
4.43	4.55	2.99	16.50	27.40	21.40	56.10	128.73	40.27	17.54
3.96	3.67	3.86	12.90	21.20	19.90	65.00	225.83	48.66	14.01
3.89	3.97	3.64	11.80	22.80	19.00	78.10	197.44	39.14	15.49
4.23	4.46	3.13	14.20	24.10	21.00	109.00	377.51	56.96	16.45
3.88	4.65	4.14	10.00	14.60	20.50	104.00	424.77	44.20	10.82
3.88	4.88	4.04	7.50	12.20	19.90	155.20	528.68	51.41	15.09
4.32	5.31	3.39	10.10	15.60	21.30	166.80	750.88	53.41	11.86
3.84	4.42	4.28	12.00	16.60	20.50	119.20	513.49	53.70	12.47
4.56	5.37	2.99	15.00	18.90	23.60	132.30	436.24	48.99	14.86
4.33	5.39	3.28	9.80	14.60	21.90	175.00	452.18	45.19	17.48
4.98	5.73	2.71	17.10	23.40	23.60	129.50	469.79	42.08	11.59
3.72	4.69	4.25	7.20	11.10	20.00	116.00	378.14	47.02	14.42
3.86	5.36	4.20	3.10	6.90	17.90	122.90	450.32	47.81	13.05
4.33	5.50	3.22	8.90	13.60	21.90	64.90	314.01	55.57	11.49
3.36	4.33	4.93	5.00	8.40	18.40	120.90	483.25	55.18	13.81
4.20	4.41	3.14	13.60	21.60	21.50	90.60	300.35	49.74	15.01
4.12	4.25	3.30	13.00	21.17	21.00	90.60	300.35	49.74	15.01
4.18	5.30	3.56	7.90	12.90	20.80	112.50	533.86	55.23	11.64
3.97	5.02	3.83	7.80	12.30	20.50	163.70	366.51	36.52	16.31
3.85	5.27	4.19	3.70	7.70	18.40	87.60	390.59	53.89	12.09
3.83	4.92	4.21	6.30	11.11	19.17	87.60	390.59	53.89	12.09
4.79	5.54	2.70	17.90	21.10	24.80	104.30	377.39	43.02	11.89
5.20	6.09	2.25	17.20	23.00	24.70	194.80	502.78	46.13	17.87
4.44	5.03	3.19	17.10	20.50	23.30	94.20	269.51	45.80	16.01
4.86	5.43	2.69	22.00	23.40	25.70	143.00	304.43	35.58	16.75
4.47	4.93	2.67	15.40	24.10	22.60	97.30	239.92	45.82	18.59
4.92	5.72	1.99	18.90	27.50	24.80	90.20	302.94	52.67	15.68
4.80	5.26	2.39	19.10	28.10	23.80	87.80	276.86	49.08	15.57
4.91	5.41	2.27	20.10	29.00	24.30	79.70	219.23	50.88	18.50
4.61	5.57	2.66	11.20	18.80	20.60	112.30	401.30	51.48	14.41
4.70	5.65	2.29	13.60	18.80	22.80	89.30	311.65	52.33	15.00
4.12	5.17	3.69	8.60	13.70	20.70	106.40	352.61	49.46	14.92
3.76	4.72	3.98	7.40	11.70	19.60	91.70	331.92	55.63	15.37

3.76	5.06	3.40	4.80	8.10	19.30	81.40	408.09	56.71	11.31
3.93	5.44	4.11	3.30	7.50	17.90	117.10	412.54	51.10	14.51
4.73	5.57	2.75	17.00	21.10	24.90	69.60	245.49	51.29	14.54
3.69	3.93	3.78	8.60	15.10	19.80	175.00	587.09	55.49	16.54
3.64	3.85	3.90	9.00	17.40	18.80	76.80	789.63	74.31	7.23
4.12	4.38	3.16	13.30	23.60	20.40	87.60	346.79	60.25	15.21
3.90	3.73	3.81	12.00	19.90	19.90	107.20	630.82	64.73	11.00
4.40	5.25	3.27	12.90	17.70	22.40	82.20	217.12	40.70	15.41
4.52	5.49	2.99	12.40	17.00	23.00	81.50	362.28	56.36	12.68
4.34	5.20	3.37	12.50	16.50	22.90	128.90	423.15	50.37	15.34
4.77	5.87	2.73	11.40	17.80	22.50	125.50	415.88	50.42	15.21
4.71	5.46	2.64	18.80	20.10	25.70	177.70	502.02	37.99	13.45
4.36	4.84	3.40	18.50	20.80	23.70	174.10	417.32	35.49	14.81
4.69	5.22	2.99	20.50	22.80	24.70	118.40	357.62	40.36	13.36
4.00	4.62	3.83	13.40	16.60	22.20	163.10	483.78	44.21	14.90
5.16	5.65	2.43	27.20	26.30	27.40	151.00	351.62	34.78	14.94
4.45	5.31	3.27	12.60	17.90	22.20	94.00	436.31	52.91	11.40
3.48	4.41	4.68	6.30	9.90	19.20	172.50	494.75	48.48	16.91
4.83	5.08	3.32	28.40	26.60	26.70	124.70	131.02	14.93	14.21
5.96	7.30	0.27	24.70	21.44	32.94	142.80	308.45	34.32	15.89
4.59	6.02	2.76	7.40	12.70	21.90	119.80	368.22	47.62	15.49
4.63	5.82	2.82	10.20	15.60	22.60	96.40	307.81	48.02	15.04
4.93	5.89	2.36	16.30	19.90	25.20	92.00	229.69	43.23	17.32
5.24	6.03	2.14	21.60	23.90	26.70	156.40	643.96	55.85	13.56
3.86	4.62	4.02	10.30	14.50	20.70	151.90	559.45	54.86	14.90
4.94	5.99	2.58	14.20	19.10	24.60	112.30	453.98	53.72	13.29
4.16	5.08	3.51	10.60	14.80	21.90	154.80	433.83	41.87	14.94
4.61	6.25	2.75	5.20	10.90	20.50	154.80	518.87	49.66	14.81
4.61	5.89	2.68	9.20	14.20	22.70	131.00	471.75	51.20	14.22
3.90	4.75	3.70	9.60	13.80	20.60	120.10	408.64	51.12	15.03
3.81	4.96	4.02	6.00	9.80	20.00	129.90	459.13	51.98	14.71
4.20	4.72	3.73	15.70	19.50	22.40	315.20	308.04	28.08	28.73
5.13	6.24	1.86	16.70	19.30	26.70	155.00	389.42	36.75	14.62
4.23	5.08	3.52	11.60	16.20	21.90	126.10	416.74	41.81	12.65
4.38	5.28	3.44	11.40	17.20	21.50	107.10	382.81	42.91	12.01
3.97	4.41	3.99	15.90	18.50	22.40	81.60	291.13	45.56	12.76
3.95	4.71	3.52	12.80	14.10	23.70	158.00	402.63	40.74	15.99
4.99	5.90	1.88	19.40	29.50	24.70	40.10	242.18	65.17	10.79
4.35	5.87	3.26	5.10	10.20	20.10	41.30	213.98	56.87	10.98
5.36	6.21	1.90	22.30	23.90	27.50	95.00	346.60	47.98	13.15
4.44	4.86	3.38	19.50	22.10	23.70	211.60	416.25	31.67	16.10
4.45	5.10	3.30	15.70	20.00	22.90	149.50	207.93	31.88	22.92
4.62	5.28	3.31	15.20	21.70	22.10	133.00	199.99	26.89	17.88
4.45	5.41	3.07	12.50	16.70	23.10	126.20	409.36	49.79	15.35
4.66	5.23	2.98	19.80	22.10	24.70	76.60	441.63	62.64	10.87
4.75	5.49	2.55	19.90	20.20	26.40	117.90	336.31	43.40	15.22
4.86	5.31	2.28	19.40	26.20	24.70	95.70	453.44	60.29	12.72

4.13	4.66	3.70	15.90	18.70	22.80	87.40	239.01	42.36	15.49
4.46	4.82	3.42	21.50	23.10	24.20	123.40	424.44	48.83	14.20
4.31	4.74	3.41	19.70	20.70	24.30	211.10	571.95	37.17	13.72
4.54	5.58	2.59	13.30	15.50	25.00	94.00	362.02	43.92	11.41
3.78	4.52	4.23	10.00	14.30	20.40	132.10	501.44	48.80	12.86
3.46	4.38	4.83	5.90	10.00	18.40	67.20	421.22	54.30	8.67
4.46	5.50	3.22	10.40	16.20	21.70	117.40	330.76	39.44	13.99
3.73	4.66	4.34	7.30	11.90	19.40	69.90	392.30	50.55	9.01
3.55	4.69	4.63	4.70	8.50	18.40	126.00	370.18	43.91	14.94
3.51	4.45	4.56	6.50	9.90	19.60	111.70	516.67	61.35	13.26
4.78	5.59	2.17	17.50	26.70	24.10	79.80	150.31	33.53	17.80
3.21	3.92	4.19	5.70	14.70	16.60	106.30	283.12	39.41	14.80
3.76	4.33	4.26	12.10	15.80	20.90	120.60	450.83	44.11	11.80
4.58	5.30	2.40	15.80	25.00	23.20	57.00	196.46	46.54	13.51
3.73	3.94	3.78	9.70	18.50	19.10	67.80	198.73	43.47	14.84
3.47	3.93	4.02	7.20	15.00	18.30	82.50	194.95	38.45	16.26
4.90	5.51	2.49	23.50	22.50	27.20	143.60	336.47	31.02	13.24
3.50	4.01	3.84	7.50	16.10	18.20	51.80	117.03	35.43	15.67
4.16	4.65	2.98	12.30	20.50	21.50	92.50	203.46	58.13	26.42
4.36	5.06	3.32	14.60	18.90	22.60	109.70	444.39	50.35	12.42
4.91	6.17	1.78	14.00	15.50	27.10	127.20	201.49	25.65	16.19
5.85	6.59	1.61	28.90	28.70	29.40	69.30	475.45	55.79	8.13
3.23	4.40	5.18	2.90	5.40	17.60	124.60	400.02	48.32	15.05
3.90	4.97	4.19	6.70	12.20	18.90	128.60	464.29	50.48	13.99
4.15	5.46	3.57	5.90	10.90	19.90	76.10	204.05	42.43	15.82
3.94	4.44	3.86	15.50	17.20	23.00	172.00	315.28	35.48	19.35
4.17	5.01	3.64	11.20	15.90	21.60	126.50	463.64	48.85	13.33
4.17	4.89	3.70	12.30	17.40	21.30	97.50	355.92	48.53	13.29
3.98	4.25	3.40	12.10	23.30	19.50	142.90	613.26	54.45	12.68
4.31	4.60	2.99	14.70	24.20	21.50	84.00	398.05	50.59	10.62
4.00	4.46	3.22	11.50	21.50	20.20	53.00	256.56	44.90	9.32
4.26	5.28	3.40	9.80	14.60	21.60	195.00	475.73	40.07	16.45
4.28	5.15	3.38	11.70	16.10	22.20	155.00	459.95	38.38	12.93
3.75	4.35	4.07	12.60	14.80	22.20	132.00	443.73	44.35	13.21
4.17	4.80	3.59	14.60	17.80	22.70	208.00	404.18	43.19	22.22
4.29	4.98	3.23	15.90	17.60	24.20	146.00	284.08	38.74	19.89
4.47	4.57	2.94	16.70	24.90	22.40	62.80	153.25	43.20	17.70
4.08	4.26	3.34	12.80	22.10	20.50	85.10	346.00	57.54	14.15
4.42	4.59	2.97	15.60	25.20	21.70	62.00	298.71	66.30	13.76
4.70	4.59	2.89	20.80	28.10	23.40	49.40	240.65	56.98	11.71
4.87	5.10	2.46	20.50	26.90	24.60	73.60	161.94	47.52	21.59
3.84	3.82	3.77	10.90	17.60	20.30	93.00	335.46	50.49	13.99
4.94	5.80	2.31	18.60	20.50	26.20	80.30	319.42	47.96	12.06
4.16	5.31	3.61	7.50	12.70	20.40	121.80	616.00	54.70	10.82
4.88	5.76	2.53	16.70	20.50	24.80	99.00	234.20	41.95	17.72
3.66	4.67	4.53	6.20	10.80	18.70	123.60	365.17	42.30	14.32
3.98	5.49	3.89	3.70	7.60	18.90	110.80	301.41	35.00	12.87

4.51	5.20	3.26	15.10	20.10	22.70	162.60	498.10	43.93	14.35
4.55	5.70	2.08	14.10	26.00	22.60	71.10	313.50	48.21	10.94
4.33	6.14	3.54	2.80	7.50	18.10	124.10	299.94	37.07	15.34
4.15	4.72	2.94	12.50	22.90	20.80	74.00	329.91	49.48	11.10
3.72	3.56	4.04	10.40	18.10	19.20	83.20	227.72	32.92	12.02
3.79	4.93	4.41	5.40	10.50	18.30	71.80	153.83	32.07	14.97
4.01	3.94	3.63	12.90	21.60	20.20	74.80	242.38	49.77	15.35
4.52	5.29	0.10	16.20	16.20	20.30	125.80	329.56	32.92	12.56
5.23	6.26	2.01	17.30	21.50	25.90	68.70	250.94	49.89	13.67
4.27	5.16	3.03	13.30	15.10	24.30	218.50	446.00	36.85	18.06
5.24	6.70	1.20	13.70	15.90	27.80	139.80	429.56	44.39	14.45
4.85	6.05	2.20	12.90	16.40	25.20	141.30	348.97	37.78	15.29
4.80	5.96	2.47	11.90	17.00	23.70	148.10	438.08	43.84	14.82
4.56	5.73	2.39	12.30	14.30	25.40	82.00	385.21	56.43	12.02
4.35	5.38	3.33	9.90	15.40	21.40	95.60	278.96	46.31	15.87
3.79	3.86	3.82	10.90	21.10	19.20	63.20	334.95	52.85	9.98
4.56	6.21	3.10	4.60	10.90	19.30	88.20	200.11	34.55	15.23
6.12	6.91	1.35	30.70	30.20	30.30	93.60	279.27	34.45	11.55
4.29	5.15	2.56	12.60	23.10	21.60	93.60	229.75	36.09	14.70
4.59	5.26	3.16	16.30	21.03	23.14	89.60	276.90	43.11	13.95
5.11	6.03	2.22	18.10	21.70	25.80	151.00	405.74	46.40	17.27
4.45	5.11	3.28	16.30	19.90	23.40	171.60	516.25	40.85	13.58
3.81	4.40	4.32	11.40	16.00	20.30	202.00	573.39	47.97	16.89
4.43	5.36	3.17	14.60	21.00	22.20	99.00	355.45	48.84	13.60
3.59	4.08	4.49	12.10	15.00	21.00	159.00	532.28	46.84	13.97
4.00	3.89	3.66	12.70	20.60	20.40	94.00	301.71	46.59	14.57
4.17	4.51	3.11	13.40	24.30	20.50	86.00	288.00	46.09	13.81
4.54	5.80	2.36	10.60	12.90	25.00	199.00	451.00	55.59	24.53
3.73	4.41	4.53	9.00	18.50	19.00	104.00	278.47	42.05	15.71
4.00	3.64	3.81	9.40	14.80	19.20	198.00	784.24	59.10	14.89
4.40	4.55	2.98	13.90	24.40	19.30	60.00	330.53	51.55	9.32
4.83	5.64	2.74	16.50	21.10	24.10	172.00	310.40	32.07	17.76
4.07	4.66	3.84	13.90	17.80	21.90	98.00	460.14	49.69	10.59
3.52	3.73	4.73	15.30	17.60	21.00	73.00	363.47	46.05	9.26
4.09	4.40	3.85	19.20	20.60	23.30	111.00	445.35	48.04	11.94
4.28	5.00	3.38	14.50	17.70	23.10	93.00	507.24	68.79	12.60
4.50	5.28	3.04	15.50	18.70	23.90	135.00	348.19	34.90	13.53
3.54	4.29	4.70	8.00	12.30	19.20	129.00	187.58	24.52	16.92
4.69	5.42	2.83	17.80	20.60	24.70	138.00	464.63	52.75	15.62
4.15	4.82	3.60	14.00	17.30	22.60	166.00	544.42	38.01	11.60
5.02	5.35	2.26	21.80	27.70	25.40	94.00	294.61	49.62	15.83
4.35	4.71	2.87	14.40	22.40	22.20	93.00	338.47	44.54	12.18
4.05	3.43	3.93	14.20	19.90	20.90	113.00	342.91	38.27	12.57
4.64	5.52	2.91	14.20	19.00	23.30	116.00	399.58	48.43	14.06
4.16	4.54	3.84	17.60	20.80	22.50	124.00	237.05	31.88	16.74
3.95	4.04	3.56	11.80	20.30	20.10	107.00	484.75	53.79	11.90
4.18	4.46	3.14	13.40	23.00	20.90	175.00	437.65	43.15	17.26

3.39	3.03	4.70	7.80	13.90	18.10	183.00	652.59	43.33	12.13
3.81	4.60	4.09	9.90	13.70	21.00	152.00	397.74	39.88	15.26

Riebalu_tie	Enenergine	Baltymai_g	Angliavanc	Riebalai_g	Energ_vert	ENDOMOR	MEZOMOR	EKTOMORI	ENDOMOR
37.42	2286.41	1.25	4.13	1.43	34.38	3	3	1	2
38.58	3936.76	1.38	5.54	1.93	45.09	2	3	1	1
33.50	2685.18	1.69	3.27	1.11	29.84	3	3	1	2
39.48	2335.29	1.37	3.02	1.27	29.05	2	3	1	1
37.87	3332.20	1.46	4.95	1.74	41.24	2	3	2	1
34.82	2597.28	1.13	4.11	1.25	32.18	2	2	2	1
29.34	2531.85	1.04	4.94	1.10	33.85	2	3	2	1
27.43	6091.14	2.91	12.04	2.51	82.42	2	3	2	1
33.18	3518.96	1.34	6.35	1.70	46.00	2	2	2	1
21.00	4441.92	1.92	9.98	1.41	60.27	2	3	2	1
37.84	2697.66	1.69	4.14	1.58	37.52	2	3	2	1
40.83	4981.70	1.34	6.03	2.26	49.82	2	3	1	1
50.00	1297.33	0.78	2.05	1.26	22.68	2	2	2	1
42.19	1278.85	0.93	2.13	0.99	21.21	2	2	1	1
37.32	1856.31	1.07	3.71	1.27	30.53	2	2	2	1
45.37	2018.01	1.51	3.82	1.97	39.03	2	2	2	1
26.59	2650.95	1.85	6.41	1.33	45.01	2	2	2	1
44.98	3843.72	1.52	6.20	2.80	56.11	2	2	2	1
33.50	4113.66	2.52	8.57	2.48	66.67	2	2	2	1
34.73	5623.62	2.58	11.62	3.36	87.05	2	3	2	1
33.84	3825.18	1.65	7.12	1.99	53.05	2	2	2	1
36.15	3561.62	1.67	5.51	1.81	44.97	2	3	1	1
37.33	4002.79	2.61	6.75	2.48	59.74	2	3	2	1
46.33	4466.09	1.77	6.42	3.14	61.01	2	3	1	1
38.56	3216.85	1.79	5.84	2.13	49.72	2	2	2	1
39.13	3767.41	2.74	10.05	3.66	84.09	2	3	2	1
32.93	2260.14	0.99	4.80	1.26	34.56	2	3	2	1
31.02	3503.24	2.03	8.12	2.03	58.88	2	2	2	1
35.25	2415.16	1.44	4.77	1.50	38.34	2	2	2	1
35.25	2415.16	1.48	4.89	1.54	39.33	2	2	2	1
33.13	3866.59	1.83	8.69	2.32	62.97	2	3	2	1
47.17	4014.48	2.57	5.76	3.31	63.12	2	3	2	1
34.02	2899.09	1.82	8.10	2.27	60.15	2	3	2	1
34.02	2899.09	1.54	6.89	1.93	51.13	2	2	2	1
45.09	3508.97	1.23	4.45	2.07	41.33	2	3	1	1
36.00	4360.04	2.60	6.71	2.33	58.21	3	3	1	2
38.19	2353.83	1.13	3.24	1.20	28.26	2	3	2	1
47.67	3422.55	1.52	3.24	1.93	36.37	2	3	1	1
35.59	2094.46	1.53	3.76	1.30	32.83	2	2	1	1
31.65	2300.90	1.31	4.40	1.18	33.44	2	3	1	1
35.34	2256.21	1.29	4.07	1.30	33.18	2	3	1	1
30.63	1723.69	1.15	3.16	0.85	24.87	2	3	1	1
34.11	3117.92	1.88	6.73	1.98	52.31	2	3	1	1
32.66	2382.04	1.24	4.32	1.20	32.99	2	3	1	1
35.62	2851.61	1.70	5.62	1.80	45.48	2	3	2	1
29.00	2386.48	1.45	5.24	1.21	37.64	2	2	2	1

31.98	2878.38	1.38	6.91	1.73	48.70	2	3	2	1
34.39	3229.47	2.66	9.38	2.80	73.40	2	3	2	1
34.17	1914.71	0.86	3.04	0.90	23.73	2	3	1	1
28.01	4232.03	3.06	10.28	2.31	74.12	2	2	2	1
18.46	4250.60	1.49	15.27	1.69	82.22	2	2	2	1
24.54	2302.46	1.54	6.08	1.10	40.39	2	2	2	1
24.26	3898.05	1.78	10.46	1.74	64.64	2	2	2	1
43.88	2133.61	1.13	2.99	1.43	29.35	2	3	2	1
30.96	2571.34	1.12	4.97	1.21	35.27	2	3	1	1
34.29	3360.61	1.70	5.58	1.69	44.28	2	3	2	1
34.37	3299.39	1.96	6.50	1.97	51.55	2	3	1	1
48.56	5285.81	1.90	5.37	3.05	56.59	2	3	1	1
49.71	4703.84	1.96	4.70	2.93	52.97	2	2	2	1
46.27	3544.14	1.32	3.98	2.03	39.42	2	3	1	1
40.89	4377.08	2.02	5.98	2.46	54.10	2	2	2	1
50.29	4044.27	1.46	3.40	2.19	39.15	3	3	1	2
35.69	3298.39	1.34	6.21	1.86	46.92	2	3	2	1
34.61	4081.70	2.71	7.78	2.47	64.18	2	2	2	1
70.86	3509.30	1.17	1.23	2.59	32.92	2	3	2	1
49.80	3595.30	1.24	2.68	1.73	31.21	3	4	1	2
36.89	3092.85	2.06	6.33	2.18	53.14	2	3	1	1
36.93	2564.01	1.48	4.72	1.61	39.33	2	3	1	1
41.19	2125.10	1.12	2.81	1.19	25.98	2	3	1	1
30.58	4611.99	1.73	7.12	1.73	51.02	3	3	1	2
30.24	4079.18	2.14	7.89	1.93	57.53	2	2	2	1
32.99	3380.55	1.51	6.09	1.66	45.38	2	3	1	1
43.19	4144.24	2.16	6.04	2.77	57.72	2	3	2	1
35.53	4179.13	3.23	10.83	3.44	87.25	2	3	1	1
34.58	3685.38	2.02	7.27	2.18	56.79	2	3	1	1
33.85	3197.51	1.73	5.87	1.73	45.94	2	2	2	1
33.32	3533.46	2.12	7.49	2.13	57.64	2	2	2	1
43.19	4388.63	3.91	3.82	2.61	54.38	2	2	2	1
48.63	4238.95	1.79	4.50	2.65	49.01	3	3	1	2
45.54	3986.91	1.76	5.82	2.82	55.68	2	3	2	1
45.08	3568.57	1.62	5.79	2.70	53.99	2	3	2	1
41.68	2556.16	0.95	3.39	1.38	29.76	2	2	2	1
43.27	3953.01	1.74	4.42	2.09	43.44	2	2	2	1
24.04	1486.40	0.61	3.69	0.60	22.62	2	3	1	1
32.15	1505.12	0.82	4.27	1.07	30.04	2	3	2	1
38.87	2889.55	1.02	3.72	1.34	31.00	3	3	1	2
52.23	5256.71	2.39	4.71	3.45	59.47	2	2	2	1
45.20	2609.18	1.91	2.65	1.67	33.28	2	3	2	1
55.23	2975.16	1.90	2.86	2.61	42.56	2	3	2	1
34.87	3288.88	1.69	5.47	1.70	43.97	2	3	2	1
26.50	2820.25	0.85	4.92	0.93	31.44	2	3	1	1
41.38	3099.54	1.20	3.42	1.45	31.53	2	3	1	1
26.99	3008.41	1.29	6.13	1.22	40.65	2	3	1	1

42.15	2256.76	1.03	2.81	1.24	26.55	2	2	2	1
36.97	3476.83	1.33	4.57	1.54	37.43	2	2	2	1
49.10	6154.20	2.22	6.00	3.52	64.58	2	2	2	1
44.67	3297.24	1.10	4.23	1.91	38.52	2	3	1	1
38.34	4109.89	1.89	7.17	2.50	58.80	2	2	2	1
37.03	3103.00	1.14	7.14	2.16	52.59	2	2	2	1
46.57	3354.69	1.83	5.16	2.71	52.34	2	3	2	1
40.44	3104.33	1.13	6.37	2.26	50.39	2	2	2	1
41.15	3372.46	2.28	6.71	2.79	61.10	2	2	2	1
25.39	3368.86	1.70	7.88	1.45	51.35	2	2	2	1
48.67	1792.86	1.22	2.29	1.48	27.37	2	3	1	1
45.80	2873.91	2.73	7.28	3.76	73.88	2	2	2	1
44.09	4088.10	1.58	5.90	2.62	53.51	2	2	2	1
39.94	1688.42	0.90	3.11	1.19	26.76	2	3	1	1
41.70	1828.74	1.29	3.79	1.61	34.83	2	2	2	1
45.28	2027.92	1.72	4.07	2.13	42.34	2	2	2	1
55.74	4338.22	1.38	3.23	2.58	41.59	2	3	1	1
48.90	1321.26	1.13	2.54	1.56	28.72	2	2	2	1
15.45	1399.97	1.54	3.39	0.40	23.33	2	2	1	1
37.23	3530.47	1.42	5.74	1.89	45.61	2	3	2	1
58.16	3142.04	1.40	2.22	2.24	34.68	2	3	1	1
36.08	3409.05	0.69	4.72	1.36	33.85	3	3	1	2
36.63	3311.21	2.32	7.44	2.50	61.55	2	2	3	1
35.53	3678.78	2.35	8.49	2.65	67.25	2	2	2	1
41.75	1923.48	1.40	3.76	1.64	35.42	2	3	2	1
45.17	3554.72	1.91	3.50	1.98	39.45	2	2	2	1
37.82	3796.21	1.79	6.57	2.26	53.77	2	3	2	1
38.18	2933.37	1.38	5.03	1.76	41.49	2	2	2	1
32.86	4505.01	2.75	11.82	3.17	86.80	2	2	2	1
38.79	3147.48	1.38	6.55	2.23	51.77	2	2	1	1
45.78	2285.50	0.99	4.79	2.17	42.64	2	2	2	1
43.49	4749.48	2.91	7.10	3.43	70.89	2	3	2	1
48.69	4793.21	2.13	6.32	3.56	65.84	2	3	2	1
42.43	4001.72	1.55	5.21	2.22	47.02	2	2	2	1
34.59	3743.68	2.53	4.92	1.75	45.60	2	2	2	1
41.37	2933.27	1.62	3.15	1.49	32.52	2	2	2	1
39.10	1419.13	0.94	2.29	0.92	21.18	2	2	1	1
28.31	2405.12	1.47	5.99	1.31	41.61	2	2	2	1
19.94	1802.10	1.00	4.82	0.64	29.07	2	2	1	1
31.31	1689.32	0.67	3.25	0.79	22.83	2	2	1	1
30.89	1363.02	0.97	2.13	0.61	17.89	2	3	1	1
35.51	2657.49	1.50	5.43	1.70	43.00	2	2	2	1
39.98	2664.16	0.89	3.53	1.31	29.41	2	3	1	1
34.48	4504.19	2.07	10.46	2.93	76.47	2	3	2	1
40.32	2233.03	1.22	2.88	1.23	27.43	2	3	1	1
43.38	3452.98	2.15	6.36	2.90	60.16	2	2	2	1
52.13	3444.23	2.29	6.23	4.12	71.16	2	3	2	1

41.72	4535.25	2.17	6.63	2.80	60.39	2	3	2	1
40.85	2601.07	1.31	5.77	2.17	47.90	2	3	1	1
47.59	3236.10	3.31	8.00	4.56	86.30	2	3	2	1
39.42	2667.00	1.35	6.03	2.14	48.76	2	2	1	1
55.05	2766.83	1.45	3.96	2.94	48.12	2	2	2	1
52.96	1918.37	1.39	2.98	2.19	37.18	2	2	2	1
34.88	1948.05	1.25	4.06	1.26	32.63	2	2	2	1
54.52	4004.51	1.57	4.12	3.04	50.12	2	3	1	1
36.44	2011.82	0.86	3.13	1.01	25.05	3	3	1	2
45.09	4840.85	2.49	5.08	2.76	55.13	2	3	2	1
41.17	3871.00	1.62	4.98	2.05	44.91	3	3	1	2
46.93	3694.75	1.79	4.43	2.44	46.89	2	3	1	1
41.34	3996.92	2.11	6.24	2.62	56.94	2	3	1	1
31.55	2730.40	0.95	4.48	1.11	31.75	2	3	1	1
37.82	2409.43	1.49	4.35	1.58	37.59	2	3	2	1
37.17	2534.92	1.17	6.18	1.93	46.77	2	2	2	1
50.22	2316.78	2.09	4.73	3.06	54.77	2	3	2	1
54.00	3242.44	0.92	2.75	1.91	31.91	3	3	1	2
49.21	2546.52	1.71	4.21	2.55	46.64	2	3	1	1
42.94	2569.13	1.16	3.57	1.58	33.15	2	3	2	1
36.33	3497.48	1.81	4.86	1.69	41.89	3	3	1	2
45.57	5055.06	2.10	6.31	3.13	61.80	2	3	2	1
35.13	4780.83	2.84	8.06	2.62	67.24	2	2	2	1
37.56	2911.13	1.42	5.11	1.75	41.83	2	3	2	1
39.19	4545.17	1.98	6.61	2.46	56.46	2	2	2	1
38.84	2590.06	1.52	4.88	1.81	41.91	2	2	2	1
40.10	2496.00	1.56	5.22	2.01	45.22	2	2	2	1
19.88	3244.00	2.43	5.50	0.88	39.56	2	3	1	1
42.24	2648.90	2.14	5.72	2.55	54.39	2	2	2	1
26.01	5307.86	3.11	12.33	2.41	83.46	2	2	2	1
39.13	2564.76	1.05	5.80	1.96	45.00	2	2	1	1
50.16	3871.04	2.20	3.97	2.76	49.57	2	3	1	1
39.73	3704.20	1.25	5.88	2.09	47.37	2	2	2	1
44.69	3157.33	0.84	4.19	1.81	36.42	2	2	2	1
40.02	3708.20	1.19	4.78	1.77	39.83	2	2	2	1
18.61	2949.65	1.14	6.20	0.75	36.06	2	2	2	1
51.58	3991.23	1.63	4.21	2.77	48.26	2	3	2	1
58.56	3060.68	1.99	2.89	3.07	47.16	2	2	2	1
31.62	3523.08	1.60	5.37	1.43	40.73	2	3	1	1
50.39	5729.86	2.06	6.75	3.98	71.00	2	2	2	1
34.56	2375.13	1.19	3.74	1.16	30.18	3	3	1	2
43.28	3039.44	1.45	5.26	2.27	47.27	2	2	1	1
49.16	3584.31	1.59	4.82	2.75	50.34	2	2	2	1
37.50	3300.04	1.55	5.34	1.84	44.12	2	3	1	1
51.37	2973.89	1.46	2.80	2.00	35.11	2	2	2	1
34.31	3604.58	1.84	8.33	2.36	61.93	2	2	2	1
39.58	4056.75	3.00	7.51	3.06	69.58	2	2	2	1

44.54	6023.87	3.27	11.65	5.32	107.57	2	2	2	1
44.86	3989.44	2.10	5.49	2.74	55.03	2	2	2	1

MEZOMOR	EKTOMORFAS_2x_GROUP
2	1
2	1
2	1
2	1
2	1
1	1
2	1
2	1
1	1
2	1
2	1
2	1
1	1
1	1
1	1
1	1
1	1
1	1
1	1
2	1
1	1
2	1
2	1
2	1
1	1
2	1
2	1
1	1
1	1
1	1
2	1
2	1
2	1
1	1
2	1
2	1
2	1
2	1
1	1
2	1
2	1
2	1
2	1
2	1
2	1
1	1

2	1
2	1
2	1
1	1
1	1
1	1
1	1
2	1
2	1
2	1
2	1
2	1
1	1
2	1
1	1
2	1
2	1
1	1
2	1
2	1
2	1
2	1
2	1
2	1
1	1
2	1
2	1
2	1
2	1
1	1
1	1
1	1
2	1
2	1
2	1
1	1
1	1
2	1
2	1
2	1
1	1
2	1
2	1
2	1
2	1
2	1
2	1

1	1
1	1
1	1
2	1
1	1
1	1
2	1
1	1
1	1
1	1
2	1
1	1
1	1
2	1
1	1
1	1
2	1
1	1
1	1
2	1
2	1
2	1
1	2
1	1
2	1
1	1
2	1
1	1
1	1
1	1
1	1
2	1
2	1
1	1
1	1
1	1
1	1
1	1
1	1
1	1
2	1
1	1
2	1
2	1
2	1
1	1
2	1

2	1
2	1
2	1
1	1
1	1
1	1
1	1
2	1
2	1
2	1
2	1
2	1
2	1
2	1
2	1
1	1
2	1
2	1
2	1
2	1
2	1
2	1
1	1
2	1
1	1
1	1
1	1
2	1
1	1
1	1
1	1
2	1
1	1
1	1
1	1
1	1
2	1
1	1
2	1
1	1
2	1
1	1
1	1
2	1
1	1
1	1
1	1

1	1
1	1
